# Construction of novel lncRNA-miRNA-mRNA ceRNA networks associated with prognosis of hepatitis C virus related hepatocellular carcinoma

**DOI:** 10.1016/j.heliyon.2022.e10832

**Published:** 2022-10-01

**Authors:** Lishi Shao, Lei Liang, Qixiang Fang, Jiaping Wang

**Affiliations:** a Department of Radiology, The Second Affiliated Hospital of Kunming Medical University, 374 Dianmian Avenue, Kunming, Yunnan 650101, PR China; b Department of Oncology, The First Affiliated Hospital of Kunming Medical University, 519 Kunzhou Road, Kunming, Yunnan 650032, PR China; c Department of Urology, The First Affiliated Hospital of the Medical College of Xi'an Jiaotong University, 277 Yanta Xi Lu, Xi 'an, Shaanxi 710061, PR China

**Keywords:** Hepatitis C virus Related hepatocellular carcinoma, Bioinformatics, Prognosis, Competitive endogenous RNA, Non-coding RNA

## Abstract

**Background:**

Hepatitis C virus (HCV) infection contribute to liver fibrosis and cirrhosis, which significantly increases the risk of hepatocellular carcinoma (HCC) development. Previous studies have demonstrated the pivotal role of competitive endogenous RNA (ceRNA) networks in tumorigenesis and cancer progression. Consequently, we herein seek to identify and evaluate the prognostic relevance of a novel ceRNA network associated with HCV-related HCC.

**Methods:**

Differentially expressed genes (DEGs) in GSE140846 dataset from GEO were identified using Network Analyst, and GO, KEGG and Reactome analyses were performed. Furthermore, a protein-protein interaction network was generated, and hub genes were detected. Hub gene expression levels, as well as those of their upstream lncRNAs and miRNAs and associated survival analyses were conducted using appropriate bioinformatics databases. Predicted target relationships were used to establish putative ceRNA networks for HCV-related HCC.

**Results:**

A total of 372 and 360 up- and down-regulated DE-mRNA were identified, which were associated with nuclear division, cell cycle, and ATPase activity. A PPI network containing 704 DE-mRNAs was constructed, and the 6 hub gene with the highest degree of connectivity were selected for subsequent analysis. We discovered that 22 miRNAs and 4 lncRNAs upstream of 11 hub gene were significantly associated with poor prognosis of HCV-related HCC, and used them to constructe a prognostic ceRNA network. Further experiments confirmed the ceRNA-regulatory relationship of BUB1-hsa-miR-193a-3p-MALAT1.

**Conclusion:**

This study provides novel insights into the lncRNA-miRNA-mRNA ceRNA network, and reveals potential lncRNA biomarkers in HCV related HCC.

## 1 Introduction

Primary liver cancer is one of the ten most common types of cancer and the second leading cause of cancer-related death [1], of which hepatocellular carcinoma (HCC) accounting for 75% of the global burden of liver cancer [2]. Approximately 71 million people worldwide are chronically infected with hepatitis C virus (HCV) [3], and HCV-associated cirrhosis is a major risk factor for HCC development [4]. The prognosis of advanced HCC is very poor, with an average expected survival of 4–6 months for patients and few effective treatment options [5]. Hence, it is critical to clarify the molecular basis of the onset and progression of HCV-related HCC to guide the appropriate treatment.

To date, many microarray-based studies to date have been conducted with the aim of understanding the mechanistic basis for HCC pathogenesis, progression, diagnosis, and prognosis [6, 7, 8]. MicroRNAs (miRNAs) are small non-coding RNAs that can repress the expression of specific mRNA at the post-transcriptional level through complementary sequence binding [9]. Long non-coding RNAs (lncRNAs) can serve as competitive miRNA targets, thereby isolating miRNAs and inhibiting their function [10]. Accumulating studies have elucidated a number of key ceRNA regulatory networks consisting of lncRNAs/miRNA/mRNA interactions that regulate tumorigenesis and progression including gastric cancer [11], breast cancer [12] and pancreatic cancer [13]. For example, Yang et al. [14] revealed that LINC01133 was downregulated in gastric cancer tissues and cell lines, and it acted as ceRNA to upregulate APC mRNA expression by sponging miR-106a-3p, which in turn inhibited metastasis of gastric cancer cells. Similarly, Zheng et al. [15] showed that FAM225A acts as a ceRNA by competitively binding miR-590-3p and miR-1275, leading to upregulation of the target ITGB3 to promote proliferation and invasion of nasopharyngeal carcinoma cells. However, the precise function of ceRNA network in HCV-related HCC remains to be further clarified.

Herein, we evaluated existing dataset from the GEO database to identify differentially expressed (DE) mRNAs, miRNAs, and lncRNAs associated with HCV-related HCC. We then performed functional enrichment and PPI analyses to understand the functional relevance of identified DE-mRNAs. Through these and associated prognostic analyses, we identified 6 hub upregulated DE-mRNAs for further analysis, and identified miRNAs and lncRNAs that predicted to regulate these targets. In so doing, we established a putative ceRNA regulatory network that provides a mechanistic basis for HCV-related HCC. The results of these analyses can be used as the basis for diagnostic biomarker or therapeutic target identification for HCV-related HCC.

## 2 Materials and methods

### 2.1 Identification of differentially expressed genes (DEGs) in microarray data

The Gene Expression Omnibus (GEO; www.ncbi.nlm.nih.gov/geo/) was retrieved for "HCV-related HCC", and the dataset with mRNA, miRNA, and lncRNA expression matrices of *Homo sapiens* was filtered, finally GSE140846 was selected for analysis. The GSE140846 dataset is a SuperSeries containing 5 pairs of non-infected normal liver and HCV-related HCC tissue for total RNA-Seq, and 3 pairs of non-infected normal liver and HCV-related HCC tissue for Small RNA-Seq. Network Analyst (https://www.networkanalyst.ca/) was used to identify DE-mRNAs, DE-miRNAs, and DE-lncRNAs when comparing control and HCC patient samples in GSE140846 dataset, using adjusted *P* value <0.05 and absolute log_2_ fold change (FC) > 1 as cutoff criteria for significance. All data for this study were obtained from the GEO database and the publication guidelines and access policies for the database were strictly followed, therefore ethical review and ethical approval from the Ethics Committee was not required for this study.

### 2.2 DEGs functional annotation

Functional enrichment analyses were performed by Gene Ontology (GO; http://geneontology.org/), Kyoto Encyclopedia of Genes and Genomes (KEGG; https://www.genome.jp/kegg/), and Reactome pathway enrichment analyses, using *Homo sapiens* as the genetic background for these analyses and *P* < 0.05 as the significance threshold.

### 2.3 Construction of HCV-related HCC associated protein-protein interaction (PPI) network

A protein-protein interaction (PPI) network was prepared using Cytoscape v. 3.4.0 with the GeneMANIA plugin, with the proteins encoded by DE-mRNAs being incorporated into this network. Gene Expression Profiling Interactive Analysis (GEPIA; http://gepia.cancer-pku.cn/) database were used to validate hub gene expression profiles, with *P* < 0.05 as the significance threshold for these analyses.

### 2.4 Construction of HCV-related HCC associated ceRNA network

The miRTarBase (versions 7.0; https://mirtarbase.cuhk.edu.cn/∼miRTarBase/miRTarBase_2022/php/index.php) and lncRNA2target (versions 2.0; http://123.59.132.21/lncrna2target/index.jsp) ádatabases were used to predict the upstream miRNA and lncRNA of hub gene, respectively. The predicted upstream miRNAs and lncRNAs were matched with DE-miRNAs and DE-lncRNAs obtained from our differential expression analysis. Further, we identified DE-miRNA/DE-mRNA and DE-miRNA/DE-lncRNA target pairs by Pearson’s correlation analysis to determine the ceRNA network. Correlations with Pearson's correlation coefficient < -2 and *P* < 0.05 were retained. Finally, HCV-related HCC associated lncRNA-miRNA-miRNA ceRNA network was prepared using Cytoscape v. 3.4.0 with the clusterMaker plugin.

### 2.5 Cell culture

Human normal hepatocytes L02 and HCC cell lines (HepG2, SK1 and Huh7) were purchased from National Collection of Authenticated Cell Cultures (Shanghai, China). All cells were cultured in dulbecco's modified eagle medium (DMEM; Lonza Group Ltd., Basel, Switzerland) containing 10% fetal bovine serum, 100 U/mL penicillin and 100 mg/mL streptomycin at 37 °C with 5% CO_2_.

### 2.6 RT-qPCR assay

TRIzol reagent (Invitrogen, Carlsbad, CA, USA) was used to extract total RNA from human normal hepatocytes and HCC cell lines. Total RNA concentration was detected by UV spectrophotometer, and RNA integrity number analysis was performed using an Agilent 2100 Bioanalyser (Agilent Technologies, Santa Clara, CA, USA) and RNA 6000 LabChip kit (Agilent Technologies) with Agilent 2100 Expert software (Agilent Technologies). RNA gel representative images of L02, HepG2, SK1 and Huh7 cells are shown in Supplementary Figure 1A. Total RNA (1 μg) was reverse transcribed to cDNA using a High-capacity RNA-to-cDNA kit (Thermo Fisher Scientific, Waltham, MA, USA). RT-qPCR was performed using SYBR Premix Ex Taq II kit (TaKaRa, Tokyo, Japan) to detect the expression of lincFOXF1, MALAT1, NORAD and SBF2-AS1 by referring to the kit instructions. Relative expression of lncRNA and miRNA was calculated using the 2^−ΔΔCt^ method with GAPDH and U6 as an internal reference, respectively. The primer sequence is as follows: lincFOXF1: 5'-TCCCGTTGCCCCTATAGACA-3' and 5'-CGCGGTTTGGGCTAATGATG-3', MALAT1: 5'-GTCATAACCAGCCTGGCAGT-3' and 5'-CGAAACATTGGCACACAGCA-3', NORAD: 5'-CGTGCCTGTACTTGTCCACT-3' and 5'-CCCTTTGCACTTTGTGCTCC-3', SBF2-AS1: 5'-ACCACAGCCAGCTGCATTAT-3' and 5'-TGTGCAAGCGCTTTATCCCT-3', GAPDH: 5'-GCAACTAGGATGGTGTGGCT-3' and 5'-TCCCATTCCCCAGCTCTCATA-3'. miR-193a-3p: 5'-CTGAGGGCTGGGTCTTTGC-3' and 5'-GCCGAGAACTGGGACTTTGT-3'. U6: 5'-CCCTTCGGGGACATCCGATA-3' and 5'-TTTGTGCGTGTCATCCTTGC-3'.

### 2.7 Dual-luciferase reporter gene assay

The 3'-untranslated regions (3'-UTR) of wild-type (WT) and mutant (MUT) BUB1 and MALAT1 bound to hsa-miR-193a-3p were amplified by PCR, respectively, and the fragments were loaded into the pMIR-REPORT luciferase microRNA expression reporter vector (Thermo Fisher Scientific). Referring to the Lipofectamine 2000 kit instructions, 0.1 μg of wild-type and mutant BUB1 and MALAT1 luciferase reporter vectors were cotransfected into HEK-293 cells with hsa-miR-193a-3p mimic (Genomeditech, Shanghai, China), respectively in HEK-293 cells (National Collection of Authenticated Cell Cultures). After 48 h of transfection, RT-qPCR was used to detect the transfection efficiency of miR-193a-3p mimic. RNA gel representative images of HEK-293 cells are shown in Supplementary Figure 1A. Subsequently, cell lysates were harvested and the luciferase activity of each group was measured with a dual-luciferase reporter assay kit (Invitrogen) according to the manufacturer's instructions. The Sanger sequencing results for BUB1-WT, BUB1-MUT, MALAT1-WT and MALAT1-MUT are shown in Supplementary Figure 1B.

## 3 Results

### 3.1 Identification of DEGs associated to HCV-related HCC

**Figure 1:**
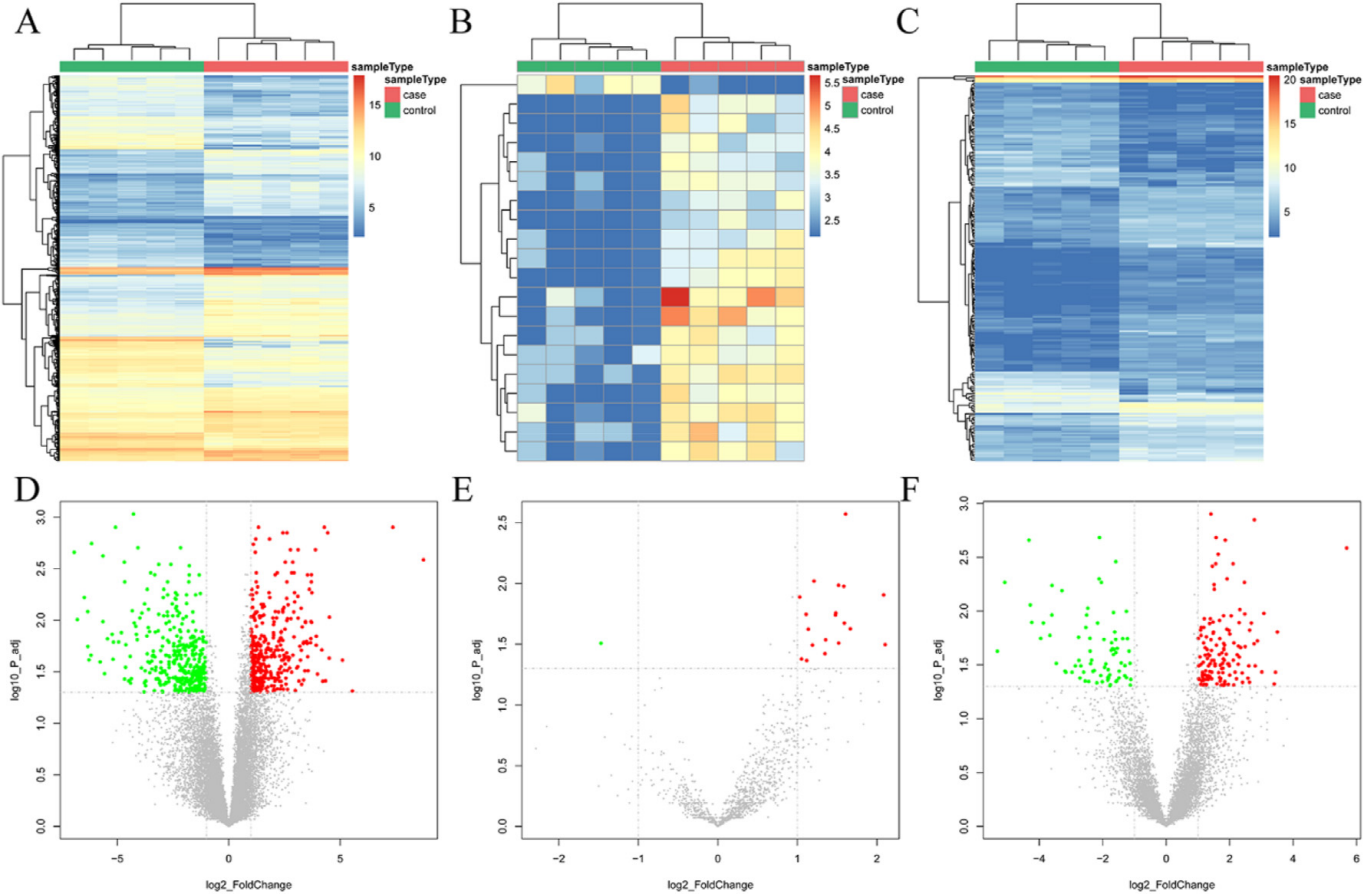
Identification of DEGs in HCV-related HCC from GEO datasets (GSE140846). (A–C) The heatmap of (A) DE-mRNAs, (B) DE-miRNAs and (C) DE-lncRNAs datasets shown by Network Analyst. Red represents the upregulated DEGs; Blue represents the downregulated DEGs. (D–F) The volcano plot of (D) DE-mRNAs, (E) DE-miRNAs, and (F) DE-lncRNAs datasets exhibited by Network Analyst. Red represents the upregulated DEGs; Blue represents the downregulated DEGs. Adjusted *P* value <0.05 and absolute log_2_ FC > 1 were the cutoff criteria for significance.

In an effort to identify key genes associated with the regulation of HCV-related HCC, we searched the GEO database and determined the GSE140846 dataset for analysis. In comparing samples from non-infected normal liver and HCV-related HCC tissue, a total of 1177 DEGs (697 up-regulated and 480 down-regulated expression) were identified, including 732 DE-mRNA (Figure 1A and Figure 1D), 20 DE-miRNA (Figure 1B and Figure 1E), and 215 DE-lncRNA (Figure 1C and Figure 1F). The top 50 DE-mRNAs and all DE-miRNAs, and DE-lncRNAs are shown in Table S Ⅰ, S Ⅱ and S Ⅲ, respectively.

### 3.2 Functional enrichment analyses of upregulated DE-mRNAs

**Figure 2:**
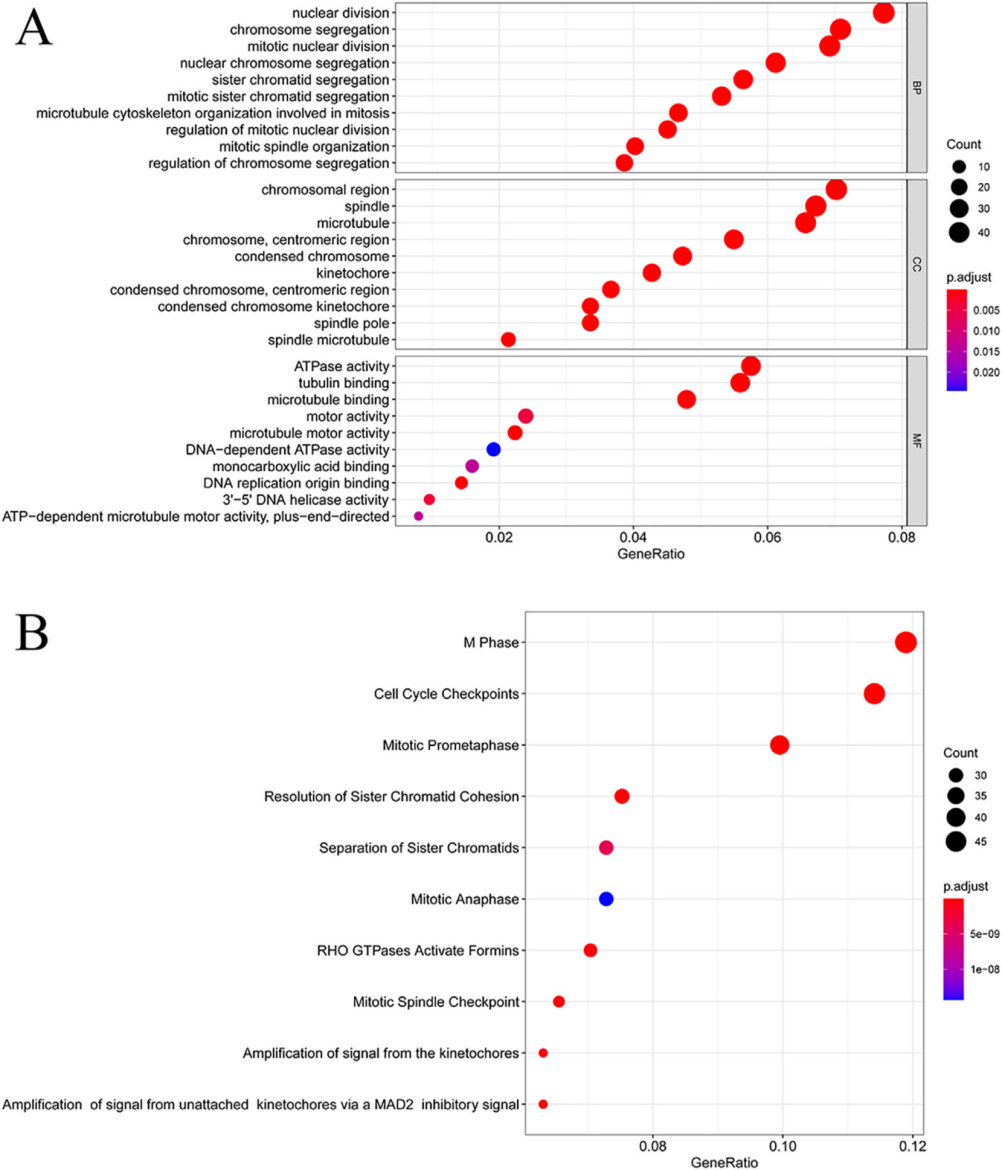
Prediction of functions and signal pathways of upregulated DE-mRNAs. (A) GO enrichment significance items of DE-mRNA in different functional groups. (B) Pathways associated with upregulated DE-mRNAs obtained by Reactome enrichment analysis. Adjusted *P* value <0.05 and absolute log_2_ FC > 1 were the cutoff criteria for significance.

The functional importance of upregulated DE-mRNAs was assessed through GO, KEGG pathway, and Reactome enrichment analyses. These results indicated that these genes were primarily associated with biological processes including nuclear division, chromosome segregation and mitotic nuclear division, cellular component including chromosomal regions, spindles and microtubule formation, and molecular functions including ATPase activity, tubulin binding and microtubule binding (Figure 2A). KEGG analyses suggested that these DE-mRNAs were significantly enriched in the cell cycle (Table S Ⅳ). Reactome enriched pathways for these DE-mRNAs included M Phase, Cell Cycle Checkpoints, and Mitotic Pro-metaphase (Figure 2B). This indicates that the upregulated DE-mRNA is most likely associated with the growth of HCV-related HCC.

### 3.3 Construction and analysis of PPI network

**Figure 3:**
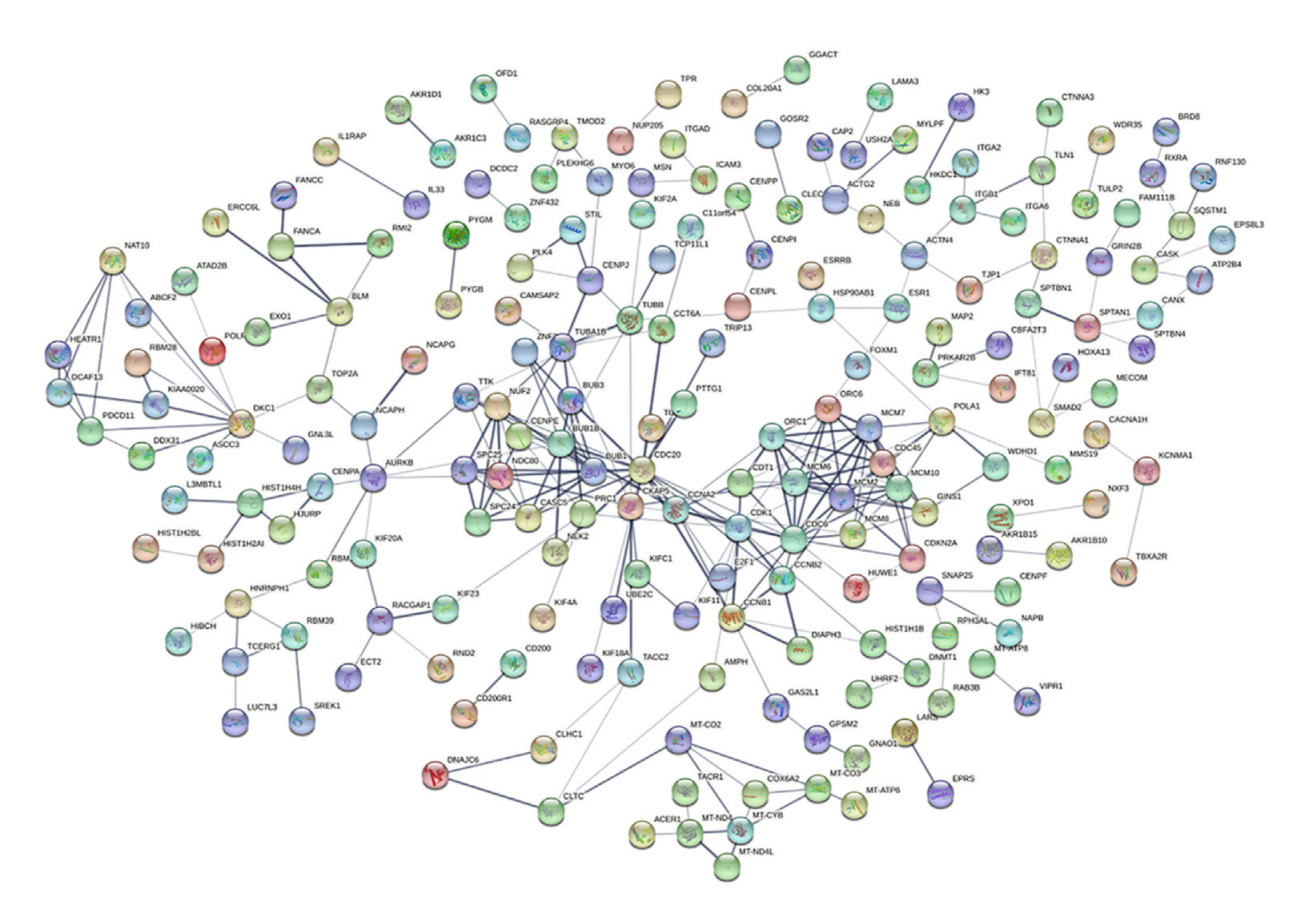
The PPI network of DE-mRNAs in HCV-related HCC.

To further explore the interactions of these DE-mRNAs in HCV-related HCC, the STRING tool was used to assess relationships among 732 DE-mRNAs in the GSE140846 dataset, resulting in the construction of a PPI network containing 704 DE-mRNAs (Figure 3). The top 11 hub genes (CDC20, CDC6, CDK1, BUB1, MCM2, BUB1B, MCM7, DKC1, NDC80, CDC45 and MCM6; Table S Ⅴ) upregulated in this network were then screened using Cytoscape for subsequent analysis. The miRNAs predicted to regulate these mRNAs were then identified using miRTarBase, and putative regulatory lncRNAs were identified with lncRNA2target, enabling the construction of a ceRNA network for these 11 hub DE-mRNAs using Cytoscape (Supplementary Figure 2). Finally, this ceRNA network includes, 62 DE-lncRNAs, 236 DE-miRNAs and 11 DE-mRNAs. (Table S Ⅵ-Ⅶ).

### 3.4 Validation of hub DE-mRNA expression and analysis of its correlation with prognosis of HCV-related HCC patients

We further verified the differential expression of 11 hub DE-mRNAs in HCC and normal samples by using the GEPIA2 database. Although not all hub gene expressions were significantly different, we found that BUB1 (Supplementary Figure 3A), CDC6 (Supplementary Figure 3C), CDC20 (Supplementary Figure 3D), CDC45 (Supplementary Figure 3E), MCM7 (Supplementary Figure 3J) and NDC80 (Supplementary Figure 3K) expressions were upregulated in HCC tumors compared with controls (*P* < 0.05). In contrast, levels of BUB1B (Supplementary Figure 3B), CDK1 (Supplementary Figure 3F), DKC1 (Supplementary Figure 3G), MCM2 (Supplementary Figure 3H), and MCM6 (Supplementary Figure 3I) did not differ between these two sample types, but there was a trend of up-regulation in the HCC tumor samples. It may be that our sample size is too small to confidently explain expression differences of these genes. We then performed survival analysis of 11 DE-mRNAs using the GEPIA2 database, and revealed that BUB1 (Supplementary Figure 3A), BUB1B (Supplementary Figure 3B), CDC20 (Supplementary Figure 3D), CDC45 (Supplementary Figure 3E), CDK1 (Supplementary Figure 3F), and NDC80 (Supplementary Figure 3K) were significantly negatively correlated with patient survival (*P* < 0.05). Therefore, we selected these 6 hub DE-mRNA for follow-up analysis.

### 3.5 Correlation analysis of upstream DE-lncRNA and DE-miRNAs of hub DE-mRNA with prognosis of HCV-related HCC

A total of 126 miRNAs predicted to regulate 6 hub DE-mRNA were identified. Next, the expression profiles of these miRNAs and the corresponding clinical information of HCC patients were downloaded from TCGA. When the 116 DE-miRNAs successfully extracted from this dataset were evaluated, those expressed in less than 100 samples were excluded, leaving 46 miRNAs for analysis. Patients were then divided into two groups based on whether the expression level of a specific miRNA was below or above the median expression level of that miRNA. The Kaplan-Meier plotter was then used to assess the prognostic relevance of these 46 miRNA. Univariate Cox regression analysis of each miRNA revealed that 22 miRNAs were significantly associated with overall survival (OS) of HCC patients. This analysis revealed that down-regulation of hsa-miR-10b-3p (Supplementary Figure 4A, *P* < 0.0001), hsa-miR-23b-3p (Supplementary Figure 4E, *P* = 0.0012), hsa-miR-192-5p (Supplementary Figure 4J, *P* = 0.00062), hsa-miR-215-5p (Supplementary Figure 4M, *P* = 0.027), hsa-miR-551b-5p (Supplementary Figure 4Q, *P* = 0.014), and hsa-miR-885-5p (Supplementary Figure 4T, *P* = 0.029) were correlated with poorer OS, whereas the up-regulation of hsa-miR-15b-5p (Supplementary Figure 4B, *P* = 0.017), hsa-miR-18a-5p (Supplementary Figure 4C, *P* < 0.0001), hsa-miR-22-3p (Supplementary Figure 4D, *P* = 0.025), hsa-miR-31-5p (Supplementary Figure 4F, *P* = 0.00091), hsa-miR-34a-5p (Supplementary Figure 4G, *P* = 0.0021), hsa-miR-186-3p (Supplementary Figure 4H, *P* = 0.0024), hsa-miR-186-5p (Supplementary Figure 4I, *P* = 0.0024), hsa-miR-193a-3p (Supplementary Figure 4K, *P* = 0.014), hsa-miR-193b-3p (Supplementary Figure 4L, *P* = 0.012), hsa-miR-301a-5p (Supplementary Figure 4N, *P* < 0.0001), hsa-miR-497-3p (Supplementary Figure 4O, *P* = 0.035), hsa-miR-497-5p (Supplementary Figure 4P, *P* = 0.035), hsa-miR-574-5p (Supplementary Figure 4R, *P* = 0.0011), hsa-miR-590-3p Supplementary Figure 4S, *P* = 0.0034), hsa-miR-935 (Supplementary Figure 4U, *P* = 0.029), and hsa-miR-1277-5p (Supplementary Figure 4V, *P* = 0.00049) was associated with poorer OS. These 22 miRNAs were considered significant prognostic miRNAs for subsequent analyses.

Next, we assessed the relevance of DE-lncRNAs regulating 6 risk DE-mRNAs to the prognosis of HCV-related HCC patients using the GEPIA2 database. A total of 4 key lncRNAs (lincFOXF1, MALAT1, NORAD and SBF2-AS1) were identified and evaluated. The results showed that high expression of lincFOXF1 (Supplementary Figure 5A, *P* = 0.000055), NORAD (Supplementary Figure 5C, *P* = 0.000057) and SBF2-AS1 (Supplementary Figure 5D, *P* = 0.0003) predicted a better prognosis for HCV-related HCC, while the opposite was true for MALAT1 (Supplementary Figure 5B, *P* = 0.0066). This suggests that 4 key DE-lncRNAs are most likely to have important regulatory roles in HCV-related HCC.

### 3.6 Construction of a ceRNA network for HCV-related HCC

**Figure 4:**
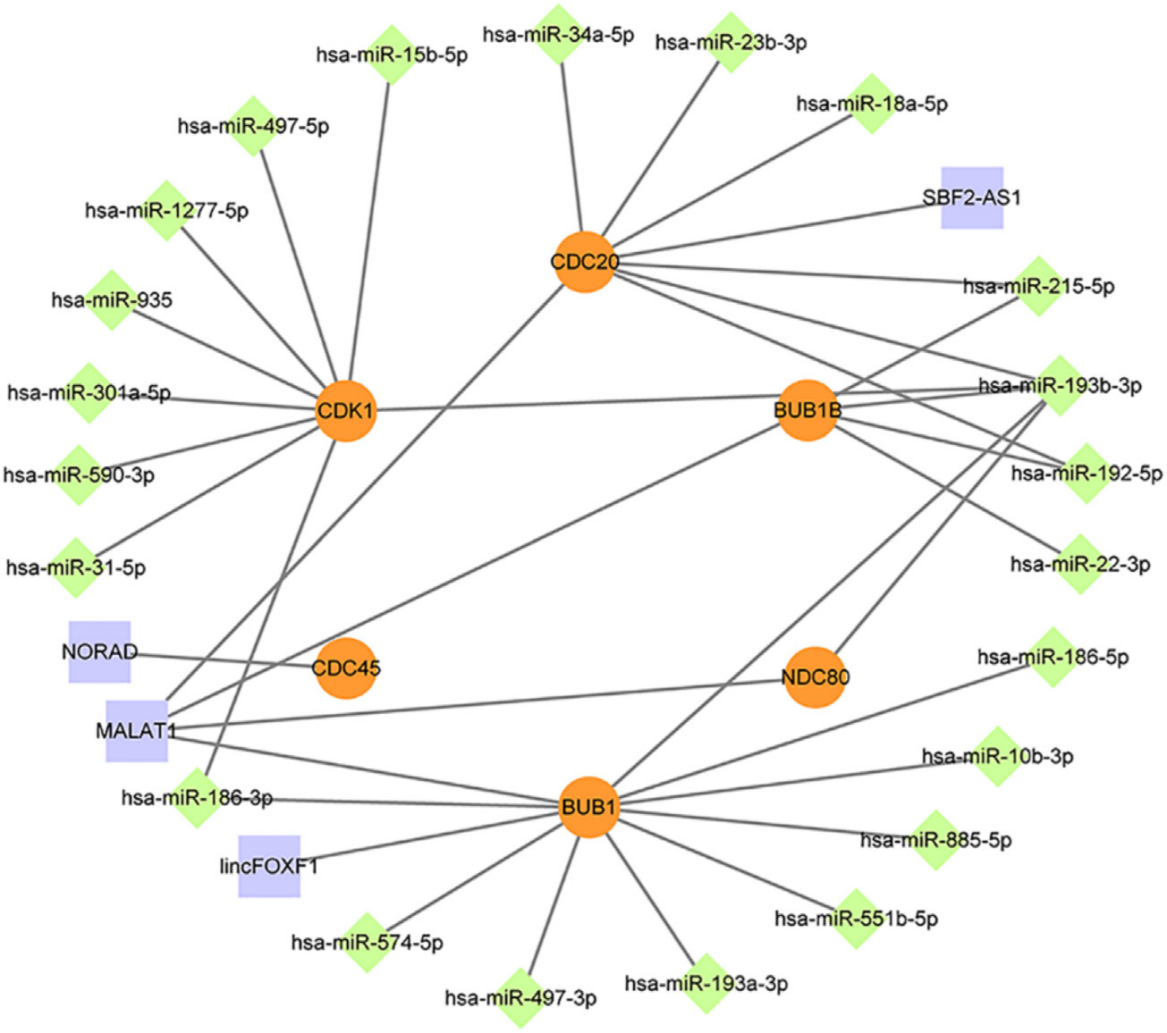
The novel lncRNA-miRNA-mRNA ceRNA network associated with prognosis of HCV-related HCC. The orange circles are mRNA, the green diamonds are miRNA and the purple squares are lncRNA.

Based on the 6 key DE-mRNAs, 22 DE-miRNAs and 4 DE-lncRNAs obtained from the screening, we next constructed an ceRNA network associated with HCV-related HCC prognosis containing 30 mRNA-miRNA pairs (CDK1-miR-15b-5p, CDK1-miR-497-5p, CDK1-miR-1277-5p, CDK1-miR-935, CDK1-miR-301a-5p, CDK1-miR-590-3p, CDK1-miR-31-5p, CDK1-miR-186-3p, CDK1-miR-193b-3p, BUB1-miR-186-3p, BUB1-miR-574-5p, BUB1-miR-497-3p, BUB1-miR-193a-3p, BUB1-miR-551b-5p, BUB1-miR-885-5p, BUB1-miR-10b-3p, BUB1-miR-186-5p, BUB1-miR-193b-3p, NDC80-miR-193b-3p, BUB1B-miR-22-3p, BUB1B-miR-192-5p, BUB1B-miR-193b-3p, BUB1B-miR-215-5p, CDC20-miR-192-5p, CDC20-miR-193b-3p, CDC20-miR-215-5p, CDC20-miR-18a-5p, CDC20-miR-23b-3p, and CDC20-miR-34a-5p) and 7 mRNA-lncRNA pairs (CDC45-NORAD, BUB1-MALAT1, BUB1-lincFOXF1, NDC80-MALAT1, BUB1B-MALAT1, CDC20-MALAT1, CDC20-SBF2-AS1), with the Cytoscape program being used for visualization (Figure 4).

As discussed above, lncRNAs can functions as ceRNAs by binding to and sequestering miRNAs, thereby indirectly upregulating miRNA target genes. Subsequently, correlations between the above mRNA/miRNA, miRNA/lncRNA and mRNA/lncRNA pairs were next evaluated to identify co-regulatory relationships consistent with ceRNA regulatory. In total, 4/90 mRNA/miRNA pairs (BUB1/BUB1B/CDC20/NDC80-hsa-miR-193a-3p), 9/24 mRNA/lncRNA pairs (BUB1/BUB1B/CDC20/CDK1/NDC80-MALAT1, BUB1/BUB1B/CDK1/NDC80-SBF2-AS1), and 3/60 miRNA-lncRNA pairs (hsa-miR-885-5p/hsa-miR-301a-5p-lincFOXF1, hsa-miR-193a-3p-MALAT1) were regulated in a manner consistent with such a ceRNA mechanism. Using these results, we identified putative mRNA/miRNA/lncRNA networks including BUB1-hsa-miR-193a-3p-MALAT1/SBF2-AS1, CDC20-hsa-miR-193a-3p-SBF2-AS1/MALAT1, BUB1B/NDC80-hsa-miR-193a-3p-SBF2-AS1, and CDK1-hsa-miR-193a-3p- MALAT1, all of which were significantly related to prognostic outcomes in HCV-related HCC patients (Table S Ⅷ-Ⅹ). After reviewing these ceRNA networks and the data shown in Figure 4, we identified BUB1-hsa-miR-193a-3p-MALAT1 as the most critical critically relevant sub-network, suggesting that it may represent a viable diagnostic or therapeutic target in HCV-related HCC.

### 3.7 Validation of lncRNA expression and BUB1-hsa-miR-193a-3p-MALAT1 ceRNA network

**Figure 5:**
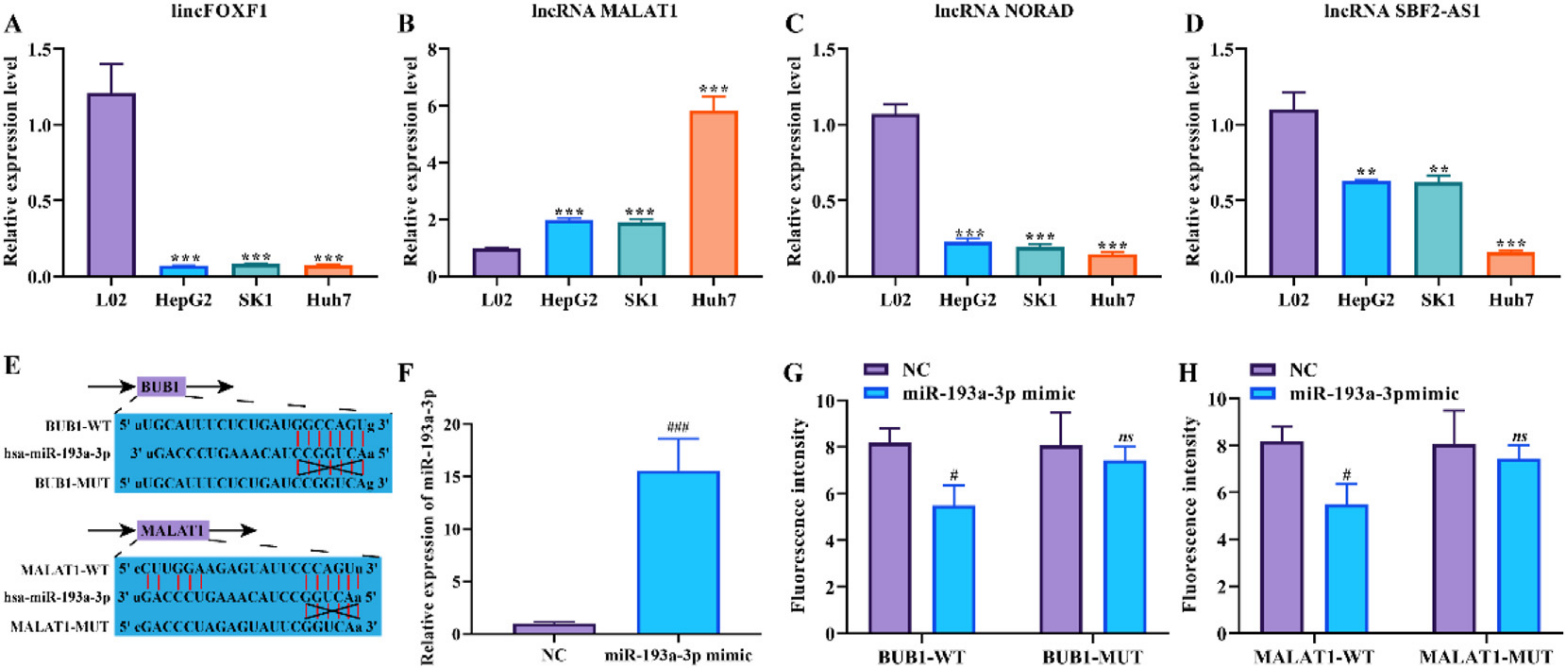
Validation of lncRNA expression and BUB1-hsa-miR-193a-3p-MALAT1 ceRNA network. (A–D) RT-qPCR was performed to verify the expression levels of (A) lincFOXF1, (B) MALAT1, (C) NORAD and (D) SBF2-AS1 in human normal hepatocyte cell line (L02) and HCC cells (HepG2, SK1 and Huh7). (E) Potential binding sequences of BUB1 and hsa-miR-193a-3p, hsa-miR-193a-3p and MALAT1, and mutant sequences of BUB1 and MALAT1. (F) The transfection efficiency of miR-193a-3p mimic in HEK-293 cells was examined by RT-qPCR. (G–H) Validation of the target binding relationship between (G) BUB1 and hsa-miR-193a-3p, (H) hsa-miR-193a-3p and MALAT1 using dual-luciferase reporter gene assays.

We further verified the expression level of 4 lncRNA in HCC cells by RT-qPCR. The results revealed that, compared to L02 cells, lincFOXF1, NORAD and SBF2-AS1 were lowly expressed in HCC cell lines (HepG2, SK1 and Huh7) (Figure 5A, C and D, *P* < 0.01), while MALAT1 was the opposite (Figure 5B, *P* < 0.001). This finding is consistent with the results of bioinformatics analysis. In addition, we verified the regulatory relationship of the BUB1-hsa-miR-193a-3p-MALAT1 ceRNA network by a dual-luciferase reporter gene assay. First, we obtained the potential binding sequences of BUB1 and hsa-miR-193a-3p, hsa-miR-193a-3p and MALAT1 through miRTarBase and lncRNA2target databases, respectively (Figure 5E). Further, we verified the targeting relationship of BUB1 and hsa-miR-193a-3p, hsa-miR-193a-3p and MALAT1 by dual-luciferase reporter gene assay. RT-qPCR results revealed that the expression of miR-193a-3p was significantly increased after transfection of miR-193a-3p mimic in HEK-293 cells (Figure 5F, *P* < 0.001). Furthermore, the fluorescence intensity of wild-type BUB1 and MALAT1 was significantly lower compared to the NC group (Figure 5G-H, *P* < 0.05), while the fluorescence intensity of mutant BUB1 and MALAT1 did not change significantly (Figure 5G-H, *P* < 0.05). It is strongly indicated that MALAT1 is most likely involved in regulating HCV-related HCC processes by acting as ceRNA regulation in the hsa-miR-193a-3p/BUB1 axis.

## 4 Discussion

The development of HCC is a complex, multi-step process, and many different putative diagnostic biomarkers for early HCC have been identified in recent years. In addition, the complex ceRNA regulatory network associated with these biomarkers has been shown to have critical prognostic relevance [16], making it an important focus of research efforts to elucidate the molecular basis of HCV-related HCC. Therefore, we herein explored viable biomarkers of this virus-associated cancer type in an effort to better aid in its diagnosis and/or treatment.

To identify hub genes associated with HCV-related HCC, we prepared a PPI networks leading to the detection of 11 key hub genes according to their degree of connectivity. These 11 DE-mRNAs were then evaluated, revealing the upregulation of BUB1, BUB1B, CDC20, CDC45, CDK1, and NDC80 to be associated with a poorer HCC patient prognosis. High Expression of BUB1 has previously been reported to be linked with poorer prognosis and increased malignancy in lymphoma, prostate, gastric, and breast cancer [17, 18, 19, 20, 21, 22, 23, 24]. BUB1B is also reported to play an oncogenic function in lung adenocarcinoma and prostate cancer [25, 26]. CDC20 upregulation has been reported in astrocytoma and gastric cancer [27], and in HCC the overexpression of this gene is associated with disease onset and progression [28]. Previous studies have suggested that CDC45 may be a new candidate gene associated with HCC risk [29, 30, 31], but the association with HCV-related HCC has not been reported. Existing studies show that NDC80 is highly expressed in HCC, and can reduce apoptosis and overcome cell cycle arrest in HCC cells [32]. Furthermore, Lu et al. [33] showed that NDC80 is an independent predictor of survival in HCC patients, and interact with NEK2 in HCC, thereby accelerating the HCC process. Hence, these DE-mRNAs are key regulators of a range of cancer types, yet the mechanisms in HCV-related HCC remain to be further elucidated.

We also evaluated the upstream DE-miRNAs of 6 hub DE-mRNAs, resulting in the identification of 116 potentially relevant regulatory miRNAs. We then assess the prognostic relevance of the high-quality miRNAs in this group, revealing 22 miRNAs to be associated with patient OS. In many cases, these miRNAs have previously been shown to function in an oncogenic or tumor suppressor capacity in other cancer types. For example, miR-10b-3p upregulation was shown to enhance HCC progression via suppressing CMTM5 expression [34], while miR-885-5p was able to target GALNT3 and to thereby inhibit PI3K/AKT/MMP signaling to disrupt intrahepatic cholangiocarcinoma metastasis [35]. Wang et al. [36] found that NSCLC patients exhibiting reduced miR-935 expression had a poorer prognosis, with this miRNA functioning by suppressing AKT pathway activation and the epithelial-mesenchymal transition of these tumor cells, impairing their *in vivo* growth. Furthermore, miR-186-5p can attenuate the metabolism of cancer cells by suppressing GLUT1 or HIF-1α expression [37, 38]. Increased miR-15b-5p has also been detected as a biomarker of more aggressive cancers [39], and miR-150-5p upregulation has previously been reported in liver cancer [40]. It is thus important the functional roles of these miRNAs in HCV-related HCC be further studied.

The role of lncRNAs as non-coding regulators of oncogenesis is well documented [41], and they serve as key biomarkers and regulators of protein translation in a range of cancers [42]. When we assessed the relative expression and prognostic relevance of DE-lncRNAs in the GSE140846 dataset using the GEPIA and LncRNA2Target platforms, we identified 4 lncRNAs potentially related to patient outcomes. Of these, lincFOXF1 had previously been shown to inhibit osteosarcoma cell invasion, migration, and metastasis *in vitro* and *in vivo* [43], whereas MALAT1 overexpression in breast cancer was found to suppress metastasis in a range of transgenic, syngeneic, and xenograft models [44]. In HCC, the MALAT1-miR195-EGFR and MALAT1 miRNA-204-SIRT1 ceRNA networks have previously been linked to tumor cell migration and invasion [45, 46]. Increased NORAD expression can inhibit the expression of miR-202-5p, thereby driving the proliferation and malignancy of thyroid carcinoma cells. The lncRNA SBF2-AS1 can drive the metastasis of HCC by controlling epithelial-mesenchymal transition, predicting a poorer patient prognosis [47]. We were able to successfully establish a lncRNA/miRNA/mRNA network for HCV-related HCC incorporating 37 regulatory relationships. Correlation analyses for RNA pairs within this network revealed the BUB1-hsa-miR-193a-3p-MALAT1 regulatory sub-network that conformed to expected ceRNA-like relationships, providing novel insight into the molecular basis for HCC onset and progression.

Notably, a large number of previous studies have explored the lncRNA/miRNA/mRNA ceRNA network in HCC [48, 49, 50, 51, 52, 53, 54, 55, 56]. In contrast to these studies, the present study is the first to investigate the lncRNA/miRNA/mRNA ceRNA network in HCV-related HCC, and the network was associated with patient prognosis. Nevertheless, there are multiple limitations to this analysis. For one, this was a bioinformatics-based approach. While this is a powerful means of exploring predicted regulatory interactions in complex disease-related contexts, the functions whereby specific miRNAs and lncRNAs function are complex and necessitate further functional research to confirm predicted ceRNA relationships and to clarify the mechanistic roles of these different targets in HCV-related HCC. While imperfect, these results nonetheless provide a foundation that can be used to predict HCV-related HCC patient outcomes and to guide the design of future *in vitro* and *in vivo* studies aimed at understanding the functional mechanisms underlying these predicted ceRNA networks.

## 5 Conclusions

In conclusion, the integrated bioinformatics analyses conducted herein led to our generation of a putative lncRNA/miRNA/mRNA ceRNA regulatory network associated with HCV-related HCC patient outcomes. These ceRNA networks offer new insights that will guide the future diagnosis and targeted treatment of this deadly disease, although additional validation will be necessary to confirm and expand upon the results of our analyses.

## Declarations

### Author contribution statement

Lishi Shao: Conceived and designed the experiments; Performed the experiments; Analyzed and interpreted the data; Wrote the paper.

Lei Liang: Performed the experiments; Analyzed and interpreted the data; Wrote the paper.

Qixiang Fang: Performed the experiments; Contributed reagents, materials, analysis tools or data; Wrote the paper.

Jiaping Wang: Conceived and designed the experiments; Performed the experiments; Wrote the paper.

### Funding statement

Dr Jiaping Wang was supported by National Natural Science Foundation of China [8186030172].

### Data availability statement

Data included in article/supp. material/referenced in article.

### Declaration of interest’s statement

The authors declare no conflict of interest.

### Additional information

No additional information is available for this paper.
