# Supplementary material for: Construction of novel lncRNA-miRNA-mRNA ceRNA networks associated with prognosis of hepatitis C virus related hepatocellular carcinoma
Source: Heliyon. 2022 Oct 1;8(10):e10832. doi: 10.1016/j.heliyon.2022.e10832 (PMC9547242; doi:10.1016/j.heliyon.2022.e10832)
Supplement: Supplementary Figure [file mmc1.docx]

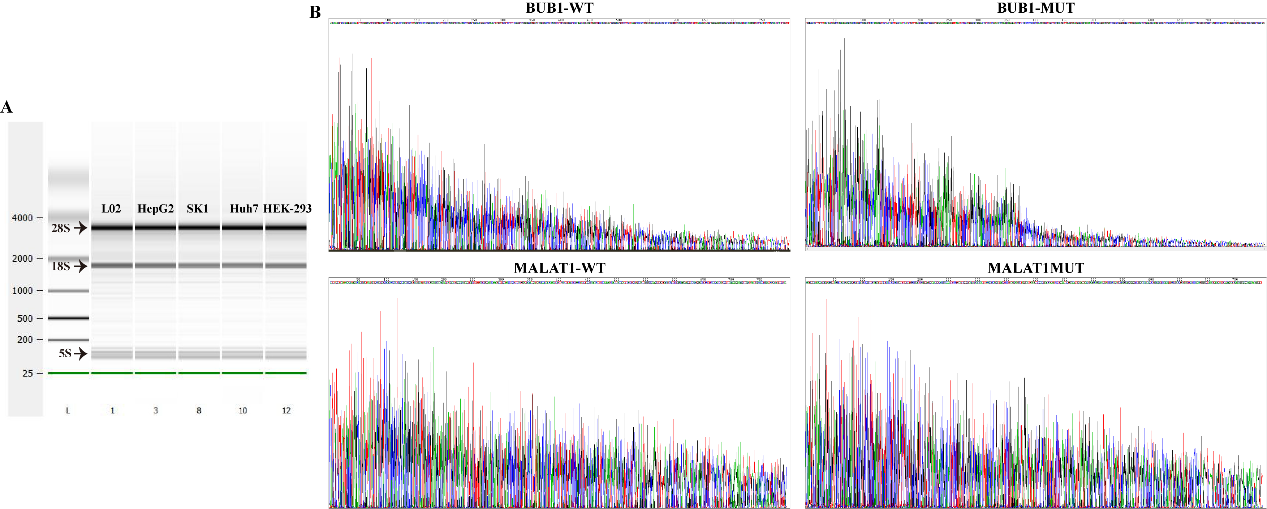


**Supplementary Figure 1.** (A) Total RNA gel representative image of L02, HepG2, SK1, Huh7 and HEK-293 cell samples. (B) Sanger sequencing results of BUB1-WT, BUB1-MUT, MALAT1-WT and MALAT1-MUT in pMIR-REPORT luciferase microRNA expression reporter vector


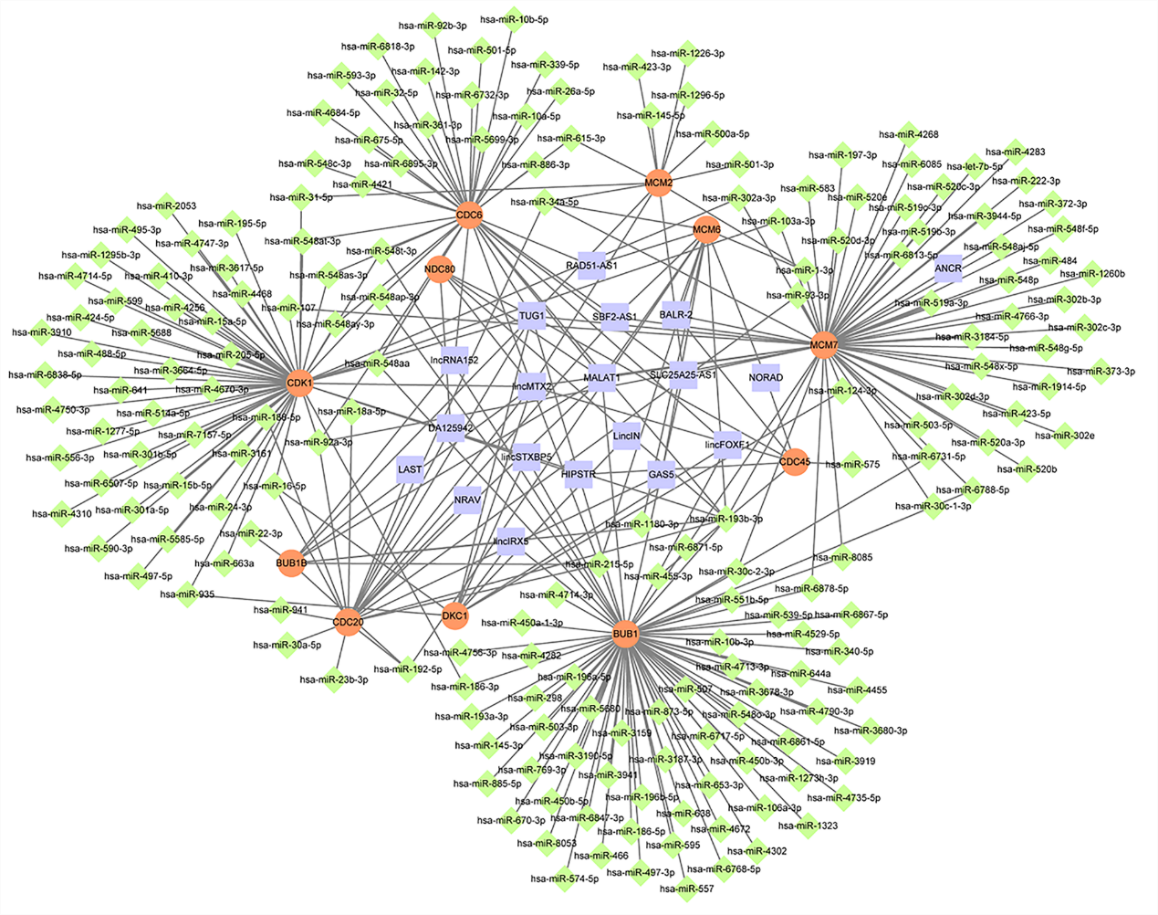


**Supplementary Figure 2. The lncRNA-miRNA-mRNA ceRNA network.** The orange circles are mRNA, the green diamonds are miRNA and the purple squares are lncRNA.


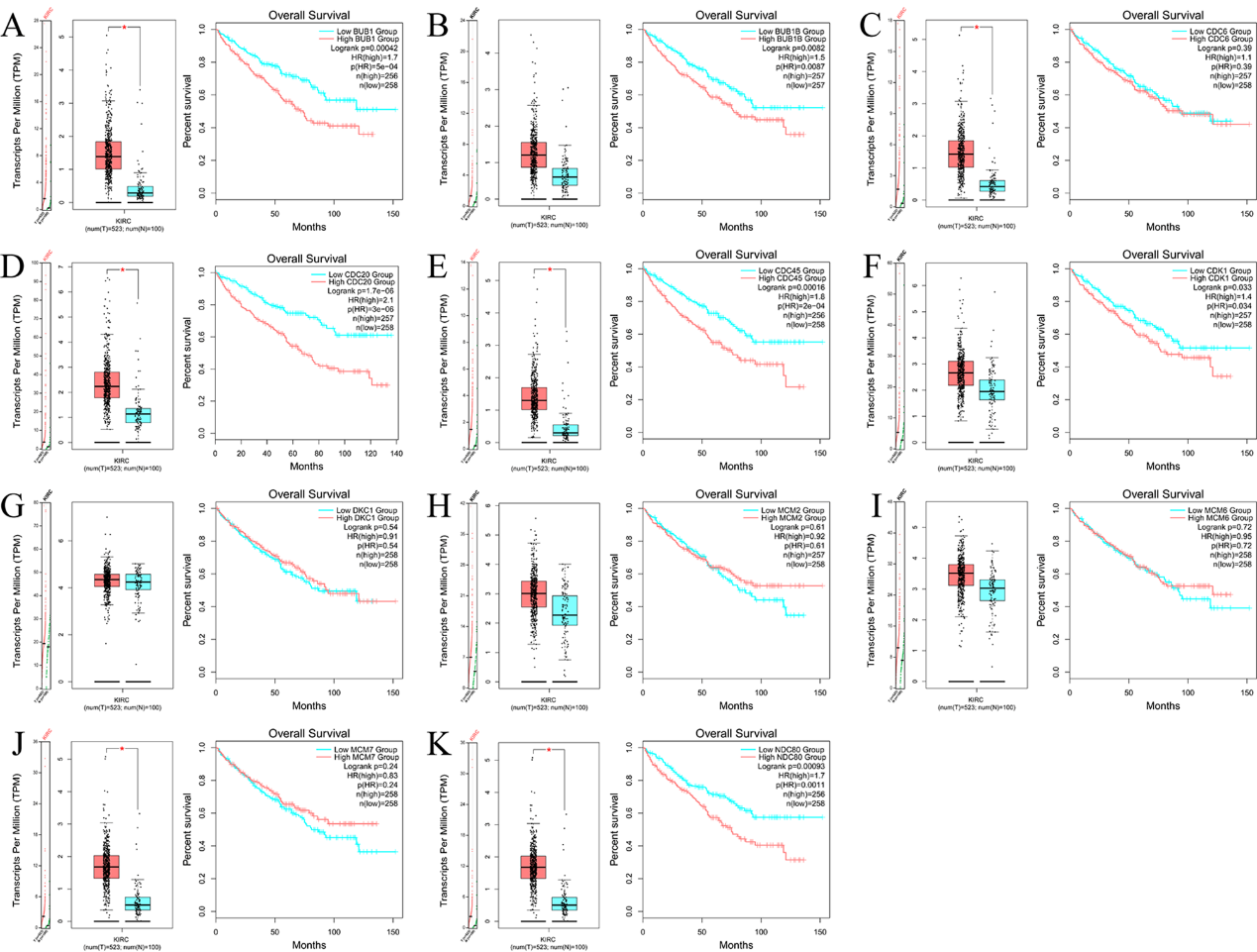


**Supplementary Figure 3. Screening for prognosis-related hub gene in patients with HCV-related HCC.** Expression (Left) and correlation with prognosis (Right) of (A) BUB1, (B) BUB1B, (C) CDC6, (D) CDC20, (E) CDC45, (F) CDK1, (G) DKC1, (H) MCM2, (I) MCM6, (J) MCM7 and (K) NDC80 in HCV-related HCC.


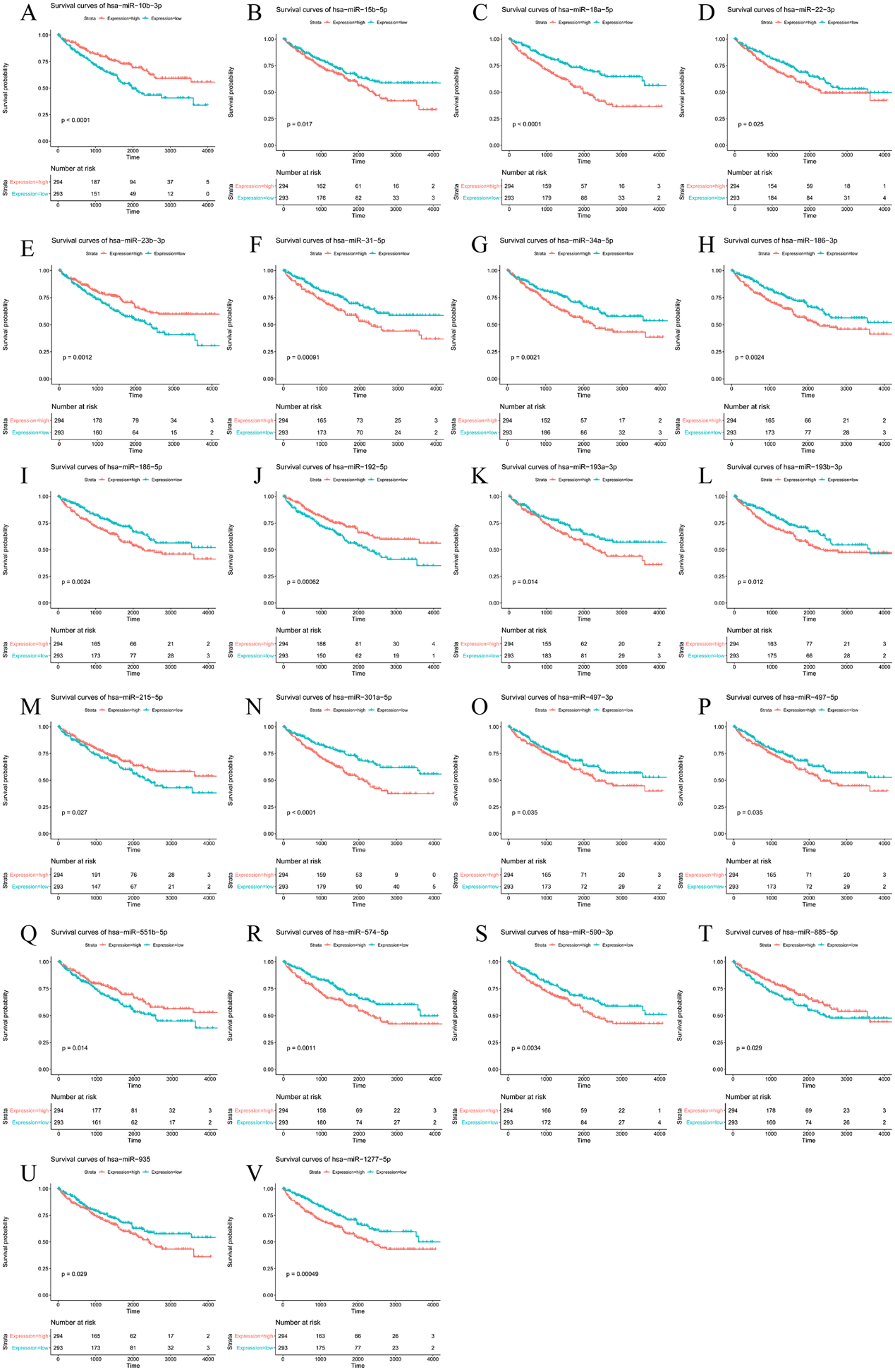


**Supplementary Figure 4. Screening for prognosis-related upstream miRNAs of hub DE-mRNA with HCV-related HCC.** Correlation with prognosis of (A) hsa-miR-10b-3p, (B) hsa−miR−15b−5p, (C) hsa−miR−18a−5p, (D) hsa−miR−22−3p, (E) hsa−miR−23b−3p, (F) hsa−miR−31−5p, (G) hsa−miR−34a−5p, (H) hsa−miR−186−3p, (I) hsa−miR−186−5p, (J) hsa−miR−192−5p, (K) hsa−miR−193a−3p, (L) hsa−miR−193b−3p, (M) hsa−miR−215−5p, (N) hsa−miR−301a−5p, (O) hsa−miR−497−3p, (P) hsa−miR−497−5p, (Q) hsa−miR−551b−5p, (R) hsa−miR−574−5p, (S) hsa−miR−590−3p, (T) hsa−miR−885−5p, (U) hsa−miR−935 and (V) hsa-miR-1277-5p in HCV-related HCC.


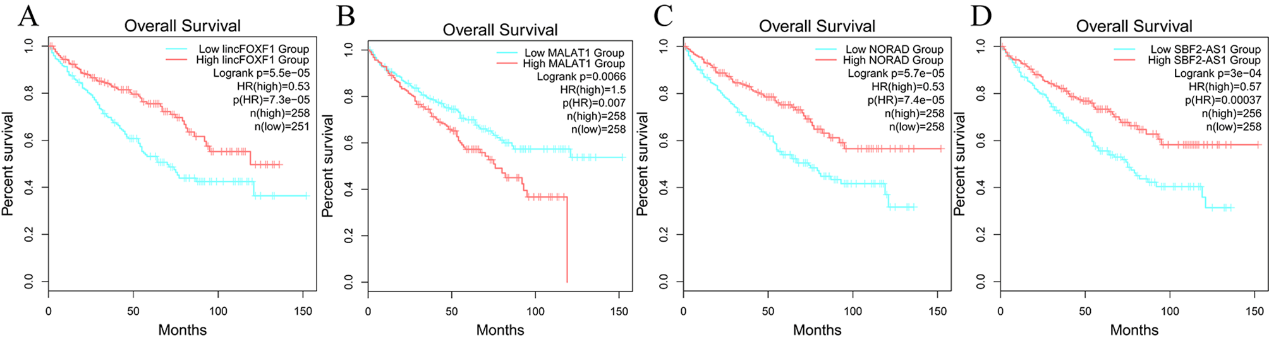


**Supplementary Figure 5. Screening for prognosis-related upstream lncRNA of hub DE-mRNA with HCV-related HCC.** Correlation with prognosis of (A) lincFOXF1, (B) MALAT1, (C) NORAD and (D) SBF2-AS1 in HCV-related HCC.
