# Supplementary material for: Construction of novel lncRNA-miRNA-mRNA ceRNA networks associated with prognosis of hepatitis C virus related hepatocellular carcinoma
Source: Heliyon. 2022 Oct 1;8(10):e10832. doi: 10.1016/j.heliyon.2022.e10832 (PMC9547242; doi:10.1016/j.heliyon.2022.e10832)
Supplement: Supplementary Tables [file mmc2.docx]

**Table S Ⅰ: List of differentially expressed mRNAs (Top 50)**

| mRNA | Fold-change | Style | Fdr | Rank |
| --- | --- | --- | --- | --- |
| PVALB | 0.051669999 | down | 0.000932776 | 1 |
| IL13RA2 | 0.029578202 | down | 0.001252636 | 2 |
| CDKN2A | 19.50990847 | up | 0.001252636 | 3 |
| MUC13 | 164.2975674 | up | 0.001252636 | 4 |
| HIST1H2BB | 2.522865168 | up | 0.001252636 | 5 |
| DTNA | 21.68831841 | up | 0.001419976 | 6 |
| HIST1H2BO | 6.114667309 | up | 0.001419976 | 7 |
| HIST1H2AI | 5.391139565 | up | 0.001419976 | 8 |
| CDC6 | 3.557898986 | up | 0.001631057 | 9 |
| CTD-3222D19.2 | 2.319028355 | up | 0.001631057 | 10 |
| IGFALS | 0.014062735 | down | 0.001801511 | 11 |
| DPH7 | 2.15129252 | up | 0.001835455 | 12 |
| DPEP3 | 0.223846895 | down | 0.001983883 | 13 |
| C14orf180 | 0.059478117 | down | 0.001983883 | 14 |
| FAT1 | 14.83920103 | up | 0.002074843 | 15 |
| PLVAP | 8.642918594 | up | 0.002074843 | 16 |
| HIST1H1B | 6.80788603 | up | 0.002074843 | 17 |
| PDCD11 | 2.284099609 | up | 0.002193481 | 18 |
| CNDP1 | 0.008186332 | down | 0.002193481 | 19 |
| KCNN2 | 0.020008511 | down | 0.002378262 | 20 |
| AKR1B10 | 426.6943164 | up | 0.002593277 | 21 |
| DTL | 7.326657597 | up | 0.002733279 | 22 |
| PCDH20 | 0.038933337 | down | 0.002733279 | 23 |
| HIST1H2AL | 5.810361589 | up | 0.002733279 | 24 |
| LTK | 0.113433819 | down | 0.002872469 | 25 |
| HMGCLL1 | 0.165586767 | down | 0.002872469 | 26 |
| MYCT1 | 0.293631 | down | 0.002963403 | 27 |
| HELLS | 7.606215333 | up | 0.003471967 | 28 |
| KIF18A | 4.517022823 | up | 0.003471967 | 29 |
| GPSM2 | 2.357661262 | up | 0.003471967 | 30 |
| MTUS2 | 0.088432806 | down | 0.003471967 | 31 |
| HIST1H3C | 7.081456587 | up | 0.003471967 | 32 |
| USH2A | 0.100197416 | down | 0.003626407 | 33 |
| DNAJC6 | 13.06737121 | up | 0.003626407 | 34 |
| AKR1C3 | 4.236440723 | up | 0.003626407 | 35 |
| ZIC2 | 11.58081687 | up | 0.003638351 | 36 |
| TUBE1 | 0.293116479 | down | 0.003638351 | 37 |
| DNAJC4 | 0.399239833 | down | 0.003638351 | 38 |
| HIST1H3B | 6.098180897 | up | 0.004254715 | 39 |
| SAT2 | 0.288953743 | down | 0.004254715 | 40 |
| CXorf36 | 13.03093807 | up | 0.004254715 | 41 |
| BSG | 2.35303884 | up | 0.004254715 | 42 |
| SLCO4C1 | 0.039419716 | down | 0.004254715 | 43 |
| KIF19 | 0.149342629 | down | 0.004254715 | 44 |
| SMAD2 | 2.454589259 | up | 0.004755241 | 45 |
| LAMA3 | 12.25699035 | up | 0.005020603 | 46 |
| PITPNM3 | 0.079266673 | down | 0.005020603 | 47 |
| ATRNL1 | 0.132691409 | down | 0.005020603 | 48 |
| CTNNA1 | 2.056300924 | up | 0.005032557 | 49 |
| ASPM | 7.732003761 | up | 0.005413619 | 50 |

**Table S Ⅱ: List of differentially expressed total miRNAs**

| microRNA | Fold-change | Style | Fdr | Rank |
| --- | --- | --- | --- | --- |
| MIR1273F | 3.045256785 | up | 0.002676386 | 1 |
| MIR4676 | 2.312275816 | up | 0.009553331 | 2 |
| MIR550A2 | 2.862434885 | up | 0.010335904 | 3 |
| AL391384.1 | 2.997455612 | up | 0.01055582 | 4 |
| AC134878.1 | 4.242515927 | up | 0.012418834 | 5 |
| AC104986.1 | 2.041876871 | up | 0.012927369 | 6 |
| AC012047.1 | 2.79522328 | up | 0.017503044 | 7 |
| AC008581.1 | 2.158720463 | up | 0.017951518 | 8 |
| MIR921 | 2.786743024 | up | 0.018178813 | 9 |
| Z85986.1 | 3.01145484 | up | 0.021220937 | 10 |
| AC118282.1 | 3.172541722 | up | 0.023617554 | 11 |
| MIR5192 | 2.200285099 | up | 0.023813156 | 12 |
| MIR1207 | 2.558364288 | up | 0.029093035 | 13 |
| MIR604 | 2.865596908 | up | 0.030964354 | 14 |
| AC009892.1 | 0.360986692 | down | 0.031068345 | 15 |
| CR392039.2 | 4.299678813 | up | 0.031884347 | 16 |
| AC104405.1 | 2.28053786 | up | 0.032173373 | 17 |
| MIR3192 | 2.543518228 | up | 0.037870422 | 18 |
| Z97351.1 | 2.073577533 | up | 0.041877751 | 19 |
| MIR1285-1 | 2.169756025 | up | 0.043137901 | 20 |

**Table S Ⅲ: List of differentially expressed lncRNAs (Top 50)**

| lncRNA | Fold-change | Style | Fdr | Rank |
| --- | --- | --- | --- | --- |
| LL0XNC01-237H1.3 | 2.653811518 | up | 0.001252636 | 1 |
| RP11-149I2.4 | 6.860048847 | up | 0.001419976 | 2 |
| CTD-2562J17.7 | 0.233197518 | down | 0.002074843 | 3 |
| Y_RNA | 2.972477521 | up | 0.002074843 | 4 |
| AC068535.3 | 0.049928005 | down | 0.002193481 | 5 |
| Y_RNA | 3.637369781 | up | 0.002193481 | 6 |
| RP11-284F21.10 | 51.52763723 | up | 0.002593277 | 7 |
| CTD-2340D6.1 | 3.113856461 | up | 0.002966977 | 8 |
| RP11-501J20.5 | 0.332883118 | down | 0.003471967 | 9 |
| RP11-168K11.2 | 2.951345586 | up | 0.003638351 | 10 |
| RP11-480A16.1 | 4.307076658 | up | 0.003638351 | 11 |
| Y_RNA | 2.741911866 | up | 0.003829869 | 12 |
| RP11-20B7.1 | 0.231126442 | down | 0.005020603 | 13 |
| CTA-217C2.2 | 3.782722221 | up | 0.005020603 | 14 |
| RP11-397G17.1 | 0.029433126 | down | 0.005413619 | 15 |
| AP000439.1 | 0.242990052 | down | 0.005413619 | 16 |
| RP11-21M24.3 | 5.522313923 | up | 0.005413619 | 17 |
| RP3-523E19.2 | 2.845303588 | up | 0.005681548 | 18 |
| RP11-830F9.5 | 0.082657352 | down | 0.005783137 | 19 |
| RP11-415J8.7 | 2.859275476 | up | 0.006266607 | 20 |
| RP11-12A2.3 | 0.102585433 | down | 0.006462556 | 21 |
| RP11-6B4.1 | 0.051405186 | down | 0.008778033 | 22 |
| AC092155.4 | 0.180344053 | down | 0.009398518 | 23 |
| RP11-70L8.4 | 4.996016194 | up | 0.009698366 | 24 |
| CTD-3162L10.4 | 0.419278609 | down | 0.010081116 | 25 |
| RP11-317J10.2 | 0.316198903 | down | 0.010314503 | 26 |
| CDKN2B-AS1 | 8.44900748 | up | 0.010520127 | 27 |
| RP4-669H2.1 | 5.565486224 | up | 0.010659431 | 28 |
| CTC-537E7.3 | 0.082385215 | down | 0.010889446 | 29 |
| RP11-598F7.3 | 0.17627409 | down | 0.010889446 | 30 |
| RP5-858B6.1 | 3.501700593 | up | 0.011055459 | 31 |
| SNHG3 | 4.130780474 | up | 0.011078728 | 32 |
| RP11-111F5.8 | 2.623708213 | up | 0.011803114 | 33 |
| CTD-2555A7.3 | 2.297711997 | up | 0.011803114 | 34 |
| BRWD1-IT1 | 3.207526763 | up | 0.012236805 | 35 |
| GS1-421I3.4 | 2.778257324 | up | 0.012654118 | 36 |
| RP11-261N11.8 | 0.053140288 | down | 0.012750142 | 37 |
| LINC00338 | 4.916852627 | up | 0.012832723 | 38 |
| RP11-284F21.7 | 6.419454969 | up | 0.012927369 | 39 |
| RP11-59E19.1 | 0.068454085 | down | 0.012927369 | 40 |
| RP11-85M11.2 | 0.223589376 | down | 0.012927369 | 41 |
| CTB-25B13.13 | 2.580380214 | up | 0.013055244 | 42 |
| KBTBD11-OT1 | 0.173035661 | down | 0.014224466 | 43 |
| RP11-568J23.5 | 2.2718731 | up | 0.014224466 | 44 |
| snoU13 | 2.148564722 | up | 0.014224466 | 45 |
| RP5-1044H5.1 | 3.76087293 | up | 0.014763429 | 46 |
| RP11-347C18.5 | 2.121705832 | up | 0.015068844 | 47 |
| SNORD3A | 6.184738453 | up | 0.01509101 | 48 |
| MIR1-2 | 3.93990374 | up | 0.015170054 | 49 |
| AC068282.3 | 0.325273853 | down | 0.015663597 | 50 |

**Table S Ⅳ: Enrichment analysis of KEGG pathway of DE-mRNAs in HCV related HCC**

| ID | Description | GeneRatio | BgRatio | pvalue | p.adjust | qvalue | Count |
| --- | --- | --- | --- | --- | --- | --- | --- |
| hsa04110 | Cell cycle | 21/270 | 124/8081 | 6.28E-10 | 1.73E-07 | 1.70E-07 | 21 |

**Table S Ⅴ: Top 11 hub genes with the highest degree in the PPI network**

| Gene Names | Full names | Degree | Description |
| --- | --- | --- | --- |
| CDC20 | Cell division cycle protein 20 homolog | 16 | Required for full ubiquitin ligase activity of the anaphase promoting complex/cyclosome (APC/C) and may confer substrate specificity upon the complex. |
| CDC6 | Cell division control protein 6 homolog | 16 | Involved in the initiation of DNA replication. Also participates in checkpoint controls that ensure DNA replication is completed before mitosis is initiated |
| CDK1 | Cyclin-dependent kinase 1 | 14 | Plays a key role in the control of the eukaryotic cell cycle by modulating the centrosome cycle as well as mitotic onset; promotes G2-M transition, and regulates G1 progress and G1-S transition via association with multiple interphase cyclins. |
| BUB1 | Mitotic checkpoint serine/threonine-protein kinase BUB1 | 12 | Serine/threonine-protein kinase that performs 2 crucial functions during mitosis: it is essential for spindle-assembly checkpoint signaling and for correct chromosome alignment. |
| MCM2 | DNA replication licensing factor MCM2 | 12 | Acts as component of the MCM2-7 complex (MCM complex) which is the putative replicative helicase essential for 'once per cell cycle' DNA replication initiation and elongation in eukaryotic cells. |
| BUB1B | Mitotic checkpoint serine/threonine-protein kinase BUB1 beta | 11 | Essential component of the mitotic checkpoint. Required for normal mitosis progression. The mitotic checkpoint delays anaphase until all chromosomes are properly attached to the mitotic spindle. |
| MCM7 | DNA replication licensing factor MCM7 | 11 | Acts as component of the MCM2-7 complex (MCM complex) which is the putative replicative helicase essential for 'once per cell cycle' DNA replication initiation and elongation in eukaryotic cells. |
| DKC1 | H/ACA ribonucleoprotein complex subunit 4 | 10 | Isoform 1: Required for ribosome biogenesis and telomere maintenance. Probable catalytic subunit of H/ACA small nucleolar ribonucleoprotein (H/ACA snoRNP) complex, which catalyzes pseudouridylation of rRNA. |
| NDC80 | Kinetochore protein NDC80 homolog | 10 | Acts as a component of the essential kinetochore- associated NDC80 complex, which is required for chromosome segregation and spindle checkpoint activity. Required for kinetochore integrity and the organization of stable microtubule binding sites in the outer plate of the kinetochore. |
| CDC45 | Cell division control protein 45 homolog | 10 | Required for initiation of chromosomal DNA replication |
| MCM6 | DNA replication licensing factor MCM6 | 10 | Acts as component of the MCM2-7 complex (MCM complex) which is the putative replicative helicase essential for 'once per cell cycle' DNA replication initiation and elongation in eukaryotic cells. |

**Table S Ⅵ: Specific mRNAs that may target specific miRNAs**

| mRNA | miRNA |
| --- | --- |
| BUB1 | hsa-miR-450a-1-3p, hsa-miR-196b-5p, hsa-miR-186-5p, hsa-miR-196a-5p, hsa-miR-10b-3p, hsa-miR-3678-3p, hsa-miR-1273h-3p, hsa-miR-8053, hsa-miR-644a, hsa-miR-6717-5p, hsa-miR-298, hsa-miR-145-3p, hsa-miR-4302, hsa-miR-769-3p, hsa-miR-450b-3p, hsa-miR-638, hsa-miR-4713-3p, hsa-miR-4735-5p, hsa-miR-3680-3p, hsa-miR-557, hsa-miR-507, hsa-miR-450b-5p, hsa-miR-6788-5p, hsa-miR-30c-2-3p, hsa-miR-30c-1-3p, hsa-miR-106a-3p, hsa-miR-8085, hsa-miR-6731-5p, hsa-miR-6878-5p, hsa-miR-4714-3p, hsa-miR-6871-5p, hsa-miR-4282, hsa-miR-670-3p, hsa-miR-340-5p, hsa-miR-503-3p, hsa-miR-6861-5p, hsa-miR-186-3p, hsa-miR-885-5p, hsa-miR-3159, hsa-miR-551b-5p, hsa-miR-3187-3p, hsa-miR-653-3p, hsa-miR-6847-3p, hsa-miR-4529-5p, hsa-miR-539-5p, hsa-miR-3190-5p, hsa-miR-4756-3p, hsa-miR-3919, hsa-miR-548o-3p, hsa-miR-1323, hsa-miR-5680, hsa-miR-193b-3p, hsa-miR-193a-3p, hsa-miR-497-3p, hsa-miR-548t-3p, hsa-miR-548ap-3p, hsa-miR-548aa, hsa-miR-4790-3p, hsa-miR-873-5p, hsa-miR-574-5p, hsa-miR-4455, hsa-miR-466, hsa-miR-4672, hsa-miR-3941, hsa-miR-6867-5p, hsa-miR-6768-5p, hsa-miR-595 |
| BUB1B | hsa-miR-193b-3p, hsa-miR-215-5p, hsa-miR-192-5p, hsa-miR-22-3p |
| CDC6 | hsa-miR-26a-5p, hsa-miR-886-3p, hsa-miR-193b-3p, hsa-miR-142-3p, hsa-miR-361-3p, hsa-miR-615-3p, hsa-miR-501-5p, hsa-miR-675-5p, hsa-miR-6818-3p, hsa-miR-4684-5p, hsa-miR-6895-3p, hsa-miR-593-3p, hsa-miR-5699-3p, hsa-miR-4421, hsa-miR-339-5p, hsa-miR-10b-5p, hsa-miR-10a-5p, hsa-miR-6732-3p, hsa-miR-548ay-3p, hsa-miR-548at-3p, hsa-miR-548as-3p, hsa-miR-548t-3p, hsa-miR-548ap-3p, hsa-miR-548aa, hsa-miR-548c-3p, hsa-miR-32-5p, hsa-miR-92b-3p, hsa-miR-92a-3p |
| CDC20 | hsa-miR-193b-3p, hsa-miR-215-5p, hsa-miR-34a-5p, hsa-miR-192-5p, hsa-miR-30a-5p, hsa-miR-16-5p, hsa-miR-941, hsa-miR-93-3p, hsa-miR-188-5p, hsa-miR-23b-3p, hsa-miR-92a-3p, hsa-miR-18a-5p |
| CDC45 | hsa-miR-455-3p, hsa-miR-575 |
| CDK1 | hsa-miR-16-5p, hsa-miR-31-5p, hsa-miR-663a, hsa-miR-590-3p, hsa-miR-193b-3p, hsa-miR-24-3p, hsa-miR-92a-3p, hsa-miR-4670-3p, hsa-miR-5585-5p, hsa-miR-301a-5p, hsa-miR-556-3p, hsa-miR-301b-5p, hsa-miR-935, hsa-miR-4256, hsa-miR-3910, hsa-miR-4714-5p, hsa-miR-6507-5p, hsa-miR-410-3p, hsa-miR-1277-5p, hsa-miR-495-3p, hsa-miR-5688, hsa-miR-302a-3p, hsa-miR-488-5p, hsa-miR-1295b-3p, hsa-miR-4747-3p, hsa-miR-548ay-3p, hsa-miR-548at-3p, hsa-miR-548as-3p, hsa-miR-548t-3p, hsa-miR-548ap-3p, hsa-miR-548aa, hsa-miR-2053, hsa-miR-4468, hsa-miR-7157-5p, hsa-miR-4310, hsa-miR-6838-5p, hsa-miR-424-5p, hsa-miR-15a-5p, hsa-miR-195-5p, hsa-miR-497-5p, hsa-miR-15b-5p, hsa-miR-186-3p, hsa-miR-599, hsa-miR-3161, hsa-miR-641, hsa-miR-3617-5p, hsa-miR-107, hsa-miR-103a-3p, hsa-miR-4750-3p, hsa-miR-205-5p, hsa-miR-3664-5p, hsa-miR-514a-5p |
| DKC1 | hsa-miR-124-3p, hsa-miR-16-5p, hsa-miR-935 |
| MCM2 | hsa-miR-1296-5p, hsa-miR-31-5p, hsa-miR-1-3p, hsa-miR-34a-5p, hsa-miR-1226-3p, hsa-miR-501-3p, hsa-miR-500a-5p, hsa-miR-615-3p, hsa-miR-423-3p, hsa-miR-145-5p |
| MCM6 | hsa-miR-193b-3p, hsa-miR-1-3p, hsa-miR-215-5p, hsa-miR-34a-5p, hsa-miR-192-5p, hsa-miR-1180-3p |
| MCM7 | hsa-miR-193b-3p, hsa-miR-124-3p, hsa-miR-1-3p, hsa-miR-34a-5p, hsa-miR-1914-5p, hsa-miR-93-3p, hsa-miR-503-5p, hsa-miR-484, hsa-miR-222-3p, hsa-miR-197-3p, hsa-miR-92a-3p, hsa-let-7b-5p, hsa-miR-1260b, hsa-miR-6788-5p, hsa-miR-30c-2-3p, hsa-miR-30c-1-3p, hsa-miR-4766-3p, hsa-miR-3944-5p, hsa-miR-4283, hsa-miR-8085, hsa-miR-6731-5p, hsa-miR-6878-5p, hsa-miR-4268, hsa-miR-107, hsa-miR-103a-3p, hsa-miR-548x-5p, hsa-miR-548g-5p, hsa-miR-548f-5p, hsa-miR-548aj-5p, hsa-miR-423-5p, hsa-miR-3184-5p, hsa-miR-6871-5p, hsa-miR-6813-5p, hsa-miR-6085, hsa-miR-548p, hsa-miR-520e, hsa-miR-520d-3p, hsa-miR-520c-3p, hsa-miR-520b, hsa-miR-520a-3p, hsa-miR-373-3p, hsa-miR-372-3p, hsa-miR-302e, hsa-miR-302d-3p, hsa-miR-302c-3p, hsa-miR-302b-3p, hsa-miR-302a-3p, hsa-miR-519c-3p, hsa-miR-519b-3p, hsa-miR-519a-3p, hsa-miR-583 |
| NDC80 | hsa-miR-193b-3p |

**Table S Ⅶ: Specific mRNAs that may target specific lncRNAs**

| mRNA | lncRNA |
| --- | --- |
| BUB1 | lincSTXBP5, SLC25A25-AS1, MALAT1, TUG1, lincFOXF1, lincIRX5, lincMTX2 |
| BUB1B | lncRNA152, MALAT1, TUG1, lincIRX5, lincMTX2 |
| CDC6 | SBF2-AS1, RAD51-AS1, SLC25A25-AS1, lncRNA152, MALAT1, lincMTX2 |
| CDC20 | LAST, SBF2-AS1, GAS5, MALAT1, NRAV, DA125942, TUG1, lincMTX2 |
| CDC45 | NORAD, HIPSTR, BALR-2, TUG1, lincMTX2 |
| CDK1 | lincMTX2 |
| DKC1 | lincSTXBP5, LincIN, TUG1, lincFOXF1, lincIRX5, lincMTX2 |
| MCM2 | SLC25A25-AS1, TUG1, lincMTX2 |
| MCM6 | SBF2-AS1, RAD51-AS1, NORAD, GAS5, MALAT1, BALR-2 |
| MCM7 | ANCR, SBF2-AS1, SLC25A25-AS1, MALAT1, BALR-2, TUG1, lincMTX2 |
| NDC80 | MALAT1, DA125942, TUG1, lincMTX2 |

**Table S Ⅷ: The correlation between mRNA-miRNA pairs identified from GSE140845 database (The pairs conformed to the ceRNA hypothesis are marked with Bold type)**

| mRNA | miRNA | R | *P*_Value |
| --- | --- | --- | --- |
| CDC45 | hsa-miR-590-3p | 0.855311568 | 0.001604147 |
| BUB1B | hsa-miR-590-3p | 0.773641826 | 0.008650684 |
| CDK1 | hsa-miR-590-3p | 0.766592652 | 0.009689828 |
| BUB1 | hsa-miR-590-3p | 0.75579003 | 0.011448492 |
| NDC80 | hsa-miR-590-3p | 0.751497681 | 0.012205634 |
| CDC20 | hsa-miR-590-3p | 0.733771647 | 0.015706108 |
| BUB1B | hsa-miR-193a-3p | -0.729805301 | 0.016575606 |
| BUB1 | hsa-miR-215-5p | 0.705639938 | 0.022607495 |
| NDC80 | hsa-miR-193a-3p | -0.682762981 | 0.02957129 |
| BUB1 | hsa-miR-193a-3p | -0.674265809 | 0.032492194 |
| CDC20 | hsa-miR-193a-3p | -0.646933623 | 0.043203824 |
| NDC80 | hsa-miR-215-5p | 0.63944097 | 0.046507403 |
| CDC20 | hsa-miR-215-5p | 0.628524926 | 0.051615938 |
| NDC80 | hsa-miR-551b-5p | -0.624145574 | 0.053765946 |
| BUB1B | hsa-miR-215-5p | 0.606523048 | 0.063015734 |
| BUB1B | hsa-miR-23b-3p | 0.605998585 | 0.063305961 |
| NDC80 | hsa-miR-23b-3p | 0.598931411 | 0.067302614 |
| BUB1 | hsa-miR-23b-3p | 0.584693378 | 0.075847226 |
| BUB1B | hsa-miR-551b-5p | -0.567561412 | 0.087023122 |
| CDC45 | hsa-miR-885-5p | 0.560540909 | 0.091891481 |
| CDC45 | hsa-miR-193a-3p | -0.557117827 | 0.094326948 |
| CDC20 | hsa-miR-1277-5p | 0.544508772 | 0.103651076 |
| BUB1 | hsa-miR-551b-5p | -0.539960824 | 0.1071517 |
| CDK1 | hsa-miR-215-5p | 0.50420838 | 0.137262228 |
| NDC80 | hsa-miR-1277-5p | 0.497055763 | 0.143846384 |
| CDC20 | hsa-miR-551b-5p | -0.490081444 | 0.150448594 |
| BUB1B | hsa-miR-1277-5p | 0.48562485 | 0.154761952 |
| CDC20 | hsa-miR-23b-3p | 0.466931712 | 0.173661232 |
| CDC45 | hsa-miR-215-5p | 0.464305388 | 0.17642126 |
| CDK1 | hsa-miR-1277-5p | 0.461659853 | 0.179227625 |
| CDC45 | hsa-miR-23b-3p | 0.456762593 | 0.184491905 |
| CDK1 | hsa-miR-193a-3p | -0.45598967 | 0.185330979 |
| BUB1 | hsa-miR-1277-5p | 0.454243469 | 0.187234889 |
| CDK1 | hsa-miR-23b-3p | 0.447261183 | 0.194962234 |
| CDK1 | hsa-miR-551b-5p | -0.419227945 | 0.227829333 |
| CDK1 | hsa-miR-574-5p | -0.370520493 | 0.291892136 |
| CDC45 | hsa-miR-1277-5p | 0.36474379 | 0.300066308 |
| CDK1 | hsa-miR-193b-3p | -0.358956688 | 0.308375588 |
| CDC45 | hsa-miR-301a-5p | 0.338906945 | 0.338084324 |
| CDC45 | hsa-miR-574-5p | -0.322192545 | 0.363922972 |
| CDK1 | hsa-miR-885-5p | 0.299994035 | 0.399701302 |
| CDC45 | hsa-miR-551b-5p | -0.26999179 | 0.450595267 |
| CDC45 | hsa-miR-193b-3p | -0.262592103 | 0.463576873 |
| BUB1B | hsa-miR-885-5p | 0.252499014 | 0.481546717 |
| BUB1 | hsa-miR-15b-5p | 0.25233581 | 0.48183974 |
| NDC80 | hsa-miR-193b-3p | -0.24151714 | 0.5014348 |
| NDC80 | hsa-miR-15b-5p | 0.228378708 | 0.525672718 |
| CDC45 | hsa-miR-186-5p | 0.21911569 | 0.543041827 |
| CDC45 | hsa-miR-186-3p | 0.21911569 | 0.543041827 |
| CDC20 | hsa-miR-574-5p | -0.217569097 | 0.545963837 |
| BUB1B | hsa-miR-186-5p | 0.21540803 | 0.550057159 |
| BUB1B | hsa-miR-186-3p | 0.21540803 | 0.550057159 |
| BUB1B | hsa-miR-193b-3p | -0.208534367 | 0.563156067 |
| CDC20 | hsa-miR-885-5p | 0.207866321 | 0.5644355 |
| BUB1 | hsa-miR-193b-3p | -0.202464087 | 0.574822475 |
| BUB1B | hsa-miR-15b-5p | 0.194061266 | 0.591119897 |
| BUB1 | hsa-miR-31-5p | -0.184602679 | 0.609663927 |
| CDC20 | hsa-miR-186-5p | 0.174314649 | 0.630063428 |
| CDC20 | hsa-miR-186-3p | 0.174314649 | 0.630063428 |
| NDC80 | hsa-miR-31-5p | -0.164639752 | 0.649455095 |
| NDC80 | hsa-miR-885-5p | 0.163062221 | 0.652635401 |
| BUB1 | hsa-miR-186-5p | 0.162390935 | 0.653990248 |
| BUB1 | hsa-miR-186-3p | 0.162390935 | 0.653990248 |
| NDC80 | hsa-miR-186-5p | 0.159663112 | 0.659505109 |
| NDC80 | hsa-miR-186-3p | 0.159663112 | 0.659505109 |
| BUB1 | hsa-miR-10b-3p | -0.159053737 | 0.660739117 |
| CDK1 | hsa-miR-31-5p | -0.156401812 | 0.666117918 |
| CDC20 | hsa-miR-193b-3p | -0.152009208 | 0.675057396 |
| CDC45 | hsa-miR-15b-5p | -0.151080144 | 0.676952892 |
| BUB1 | hsa-miR-885-5p | 0.147774322 | 0.68371069 |
| BUB1B | hsa-miR-301a-5p | 0.14565234 | 0.688059183 |
| CDC20 | hsa-miR-31-5p | -0.143041955 | 0.693419842 |
| NDC80 | hsa-miR-10b-3p | -0.137860494 | 0.704096598 |
| CDK1 | hsa-miR-301a-5p | 0.137413154 | 0.705020587 |
| CDK1 | hsa-miR-186-5p | 0.132548519 | 0.715090802 |
| CDK1 | hsa-miR-186-3p | 0.132548519 | 0.715090802 |
| CDC20 | hsa-miR-15b-5p | 0.120519942 | 0.740158717 |
| BUB1 | hsa-miR-574-5p | -0.118120769 | 0.745185916 |
| CDC20 | hsa-miR-301a-5p | 0.117405043 | 0.746687328 |
| BUB1B | hsa-miR-31-5p | -0.106134088 | 0.770429302 |
| BUB1B | hsa-miR-574-5p | -0.096831234 | 0.790156597 |
| CDK1 | hsa-miR-10b-3p | -0.094078077 | 0.796015987 |
| NDC80 | hsa-miR-574-5p | -0.090441218 | 0.803770165 |
| BUB1B | hsa-miR-10b-3p | -0.087051048 | 0.811012296 |
| CDC20 | hsa-miR-10b-3p | -0.064144258 | 0.860260339 |
| NDC80 | hsa-miR-301a-5p | 0.043141343 | 0.905803759 |
| BUB1 | hsa-miR-301a-5p | 0.037445325 | 0.918203107 |
| CDK1 | hsa-miR-15b-5p | -0.035426123 | 0.922602538 |
| CDC45 | hsa-miR-10b-3p | 0.0172405 | 0.962297613 |
| CDC45 | hsa-miR-31-5p | 0.007535298 | 0.983517472 |

**Table S Ⅸ: The correlation between mRNA-lncRNA pairs identified from GSE140845 database (The pairs conformed to the ceRNA hypothesis are marked with Bold type)**

| mRNA | lncRNA | R | P_Value |
| --- | --- | --- | --- |
| BUB1B | MALAT1 | 0.843237321 | 0.002176816 |
| NDC80 | MALAT1 | 0.817977948 | 0.003831056 |
| BUB1B | SBF2-AS1 | 0.785967865 | 0.00702689 |
| BUB1B | NORAD | -0.781992437 | 0.007524635 |
| CDC20 | SBF2-AS1 | 0.77718814 | 0.008158889 |
| BUB1 | MALAT1 | 0.765280304 | 0.009892562 |
| CDC20 | MALAT1 | 0.762123492 | 0.010392441 |
| NDC80 | SBF2-AS1 | 0.750583729 | 0.012371267 |
| BUB1 | SBF2-AS1 | 0.730088043 | 0.016512541 |
| CDC20 | NORAD | -0.718577322 | 0.019217577 |
| NDC80 | NORAD | -0.707617699 | 0.022064503 |
| CDC45 | NORAD | -0.702556137 | 0.023472399 |
| BUB1 | NORAD | -0.670753843 | 0.033754617 |
| CDK1 | MALAT1 | 0.651403064 | 0.041310174 |
| CDC45 | lincFOXF1 | -0.639342324 | 0.046551988 |
| CDC45 | MALAT1 | 0.616665732 | 0.057573848 |
| CDC45 | SBF2-AS1 | 0.597478368 | 0.068144278 |
| CDK1 | lincFOXF1 | -0.577541697 | 0.080392226 |
| CDK1 | SBF2-AS1 | 0.557751324 | 0.093873158 |
| CDK1 | NORAD | -0.53933666 | 0.10763786 |
| BUB1B | lincFOXF1 | -0.405827547 | 0.244579324 |
| CDC20 | lincFOXF1 | -0.403478374 | 0.247584574 |
| BUB1 | lincFOXF1 | -0.399728988 | 0.252423514 |
| NDC80 | lincFOXF1 | -0.379606347 | 0.27928007 |

**Table S Ⅹ: The correlation between miRNA-lncRNA pairs identified from GSE140845 database (The pairs conformed to the ceRNA hypothesis are marked with Bold type)**

| miRNA | lncRNA | R | P_Value |
| --- | --- | --- | --- |
| hsa-miR-193b-3p | lincFOXF1 | 0.700809996 | 0.023972087 |
| hsa-miR-885-5p | lincFOXF1 | -0.672504147 | 0.033121365 |
| hsa-miR-193a-3p | MALAT1 | -0.661898639 | 0.037084481 |
| hsa-miR-301a-5p | lincFOXF1 | -0.645783433 | 0.043700394 |
| hsa-miR-551b-5p | MALAT1 | -0.612841458 | 0.059587736 |
| hsa-miR-1277-5p | SBF2-AS1 | 0.609501611 | 0.061384028 |
| hsa-miR-590-3p | NORAD | -0.598668366 | 0.067454475 |
| hsa-miR-1277-5p | MALAT1 | 0.595274509 | 0.069433917 |
| hsa-miR-186-5p | NORAD | -0.585685125 | 0.075230428 |
| hsa-miR-186-3p | NORAD | -0.585685125 | 0.075230428 |
| hsa-miR-23b-3p | MALAT1 | 0.574092352 | 0.082645758 |
| hsa-miR-590-3p | lincFOXF1 | -0.568492801 | 0.086389968 |
| hsa-miR-590-3p | MALAT1 | 0.541024291 | 0.106326557 |
| hsa-miR-186-5p | SBF2-AS1 | 0.531851562 | 0.113576312 |
| hsa-miR-186-3p | SBF2-AS1 | 0.531851562 | 0.113576312 |
| hsa-miR-23b-3p | lincFOXF1 | -0.495218165 | 0.145568453 |
| hsa-miR-193a-3p | SBF2-AS1 | -0.484153827 | 0.156201916 |
| hsa-miR-590-3p | SBF2-AS1 | 0.479840288 | 0.160470866 |
| hsa-miR-193a-3p | NORAD | 0.466858222 | 0.173738111 |
| hsa-miR-10b-3p | NORAD | -0.429649293 | 0.215266824 |
| hsa-miR-15b-5p | lincFOXF1 | 0.402754872 | 0.248514263 |
| hsa-miR-23b-3p | NORAD | -0.392145278 | 0.26237005 |
| hsa-miR-15b-5p | SBF2-AS1 | 0.389943181 | 0.265298005 |
| hsa-miR-215-5p | SBF2-AS1 | 0.381088998 | 0.277250534 |
| hsa-miR-193a-3p | lincFOXF1 | 0.379436773 | 0.279512703 |
| hsa-miR-15b-5p | MALAT1 | 0.37850451 | 0.280793523 |
| hsa-miR-551b-5p | SBF2-AS1 | -0.364298502 | 0.300701396 |
| hsa-miR-186-5p | MALAT1 | 0.361407095 | 0.304842566 |
| hsa-miR-186-3p | MALAT1 | 0.361407095 | 0.304842566 |
| hsa-miR-10b-3p | lincFOXF1 | 0.360761441 | 0.305771389 |
| hsa-miR-193b-3p | SBF2-AS1 | 0.359460446 | 0.30764751 |
| hsa-miR-31-5p | NORAD | -0.323413729 | 0.362002713 |
| hsa-miR-10b-3p | SBF2-AS1 | 0.308416284 | 0.385933885 |
| hsa-miR-215-5p | MALAT1 | 0.295565435 | 0.407033532 |
| hsa-miR-551b-5p | NORAD | 0.265339 | 0.458738603 |
| hsa-miR-885-5p | NORAD | -0.251203835 | 0.483874269 |
| hsa-miR-574-5p | lincFOXF1 | 0.249695972 | 0.486590149 |
| hsa-miR-31-5p | lincFOXF1 | 0.248653957 | 0.4884708 |
| hsa-miR-215-5p | lincFOXF1 | -0.235394246 | 0.512671234 |
| hsa-miR-301a-5p | MALAT1 | 0.229127285 | 0.524279053 |
| hsa-miR-215-5p | NORAD | -0.228025748 | 0.526330367 |
| hsa-miR-193b-3p | MALAT1 | -0.216141507 | 0.548666514 |
| hsa-miR-186-5p | lincFOXF1 | 0.205241389 | 0.569473497 |
| hsa-miR-186-3p | lincFOXF1 | 0.205241389 | 0.569473497 |
| hsa-miR-193b-3p | NORAD | -0.191001408 | 0.597096229 |
| hsa-miR-23b-3p | SBF2-AS1 | 0.178337338 | 0.622059337 |
| hsa-miR-1277-5p | NORAD | -0.17095156 | 0.636781835 |
| hsa-miR-574-5p | NORAD | 0.162519323 | 0.653731055 |
| hsa-miR-885-5p | MALAT1 | 0.12289442 | 0.73519197 |
| hsa-miR-574-5p | SBF2-AS1 | -0.10121163 | 0.780853633 |
| hsa-miR-31-5p | SBF2-AS1 | 0.069902468 | 0.847833343 |
| hsa-miR-31-5p | MALAT1 | -0.046924897 | 0.897577515 |
| hsa-miR-15b-5p | NORAD | -0.04190352 | 0.908496834 |
| hsa-miR-574-5p | MALAT1 | -0.041183515 | 0.910063705 |
| hsa-miR-885-5p | SBF2-AS1 | -0.038036033 | 0.916916448 |
| hsa-miR-1277-5p | lincFOXF1 | 0.036939639 | 0.919304711 |
| hsa-miR-551b-5p | lincFOXF1 | 0.032194683 | 0.929647081 |
| hsa-miR-10b-3p | MALAT1 | -0.026709211 | 0.941615264 |
| hsa-miR-301a-5p | SBF2-AS1 | -0.02534274 | 0.944598347 |
| hsa-miR-301a-5p | NORAD | -0.004610762 | 0.989914173 |
